# Characterization of d-xylose reductase, XyrB, from *Aspergillus niger*

**DOI:** 10.1016/j.btre.2021.e00610

**Published:** 2021-03-15

**Authors:** Agata Terebieniec, Tania Chroumpi, Adiphol Dilokpimol, Maria Victoria Aguilar-Pontes, Miia R. Mäkelä, Ronald P. de Vries

**Affiliations:** a Fungal Physiology, Westerdijk Fungal Biodiversity Institute and Fungal Molecular Physiology, Utrecht University, Utrecht, the Netherlands; b Department of Molecular Biotechnology and Microbiology, Faculty of Chemistry, Gdansk University of Technology, Gdansk, Poland; c Department of Microbiology, University of Helsinki, Helsinki, Finland

**Keywords:** AKR, aldo-keto reductase, *Ct*XR, *Candida tenuis* xylose reductase, GCY1/YPR1, yeast glycerol dehydrogenases, GldB, filamentous fungal glycerol dehydrogenase, IPTG, isopropyl β-D-1-thiogalactopyranoside, LadA, l-arabitol dehydrogenase, LarA, l-arabinose reductase, LB, Luria Bertani, LxrA, LxrB, l-xylulose reductase, NAD, nicotinamide adenine dinucleotide, NADP, nicotinamide adenine dinucleotide phosphate, NADPH, reduced, PBS, phosphate buffered saline, PCP, Pentose Catabolic Pathway, PCR, polymerase chain reaction, PPP, Pentose Phosphate Pathway, PRD1, pentose reductase, SdhA, sorbitol dehydrogenase, XdhA, xylitol dehydrogenase, XkiA, d-xylulose kinase, XyrA, XyrB, d-xylose reductase, *Aspergillus niger*, d-xylose reductase, Pentose Catabolic Pathway

## Abstract

d-xylose reductase is a member of the aldo-keto reductase family, and is involved in d-xylose and l-arabinose conversion through the Pentose Catabolic Pathway (PCP) in fungi. In this study, we biochemically characterized a newly identified second d-xylose reductase (XyrB) from *Aspergillus niger*. This NADPH-dependent reductase is able to efficiently convert d-xylose and l-arabinose, and it has the highest affinity for these sugars of all currently known fungal pentose reductases. A combination of biochemical data, transcriptomics and phylogenetic analysis further illustrated the role of XyrB in the PCP.

Enzymes: D-xylose reductase (EC 1.1.1.307), L-arabinose reductase (EC 1.1.1.21).

## Highlights

• XyrB is involved in conversion of d-xylose and l-arabinose in *A. niger*.
• The *xyrB* expression is induced both by d-xylose and l-arabinose.
• *XyrB* expression is controlled by *xlnR* and *araR* regulators.

## 1 Introduction

**Fig. 1:**
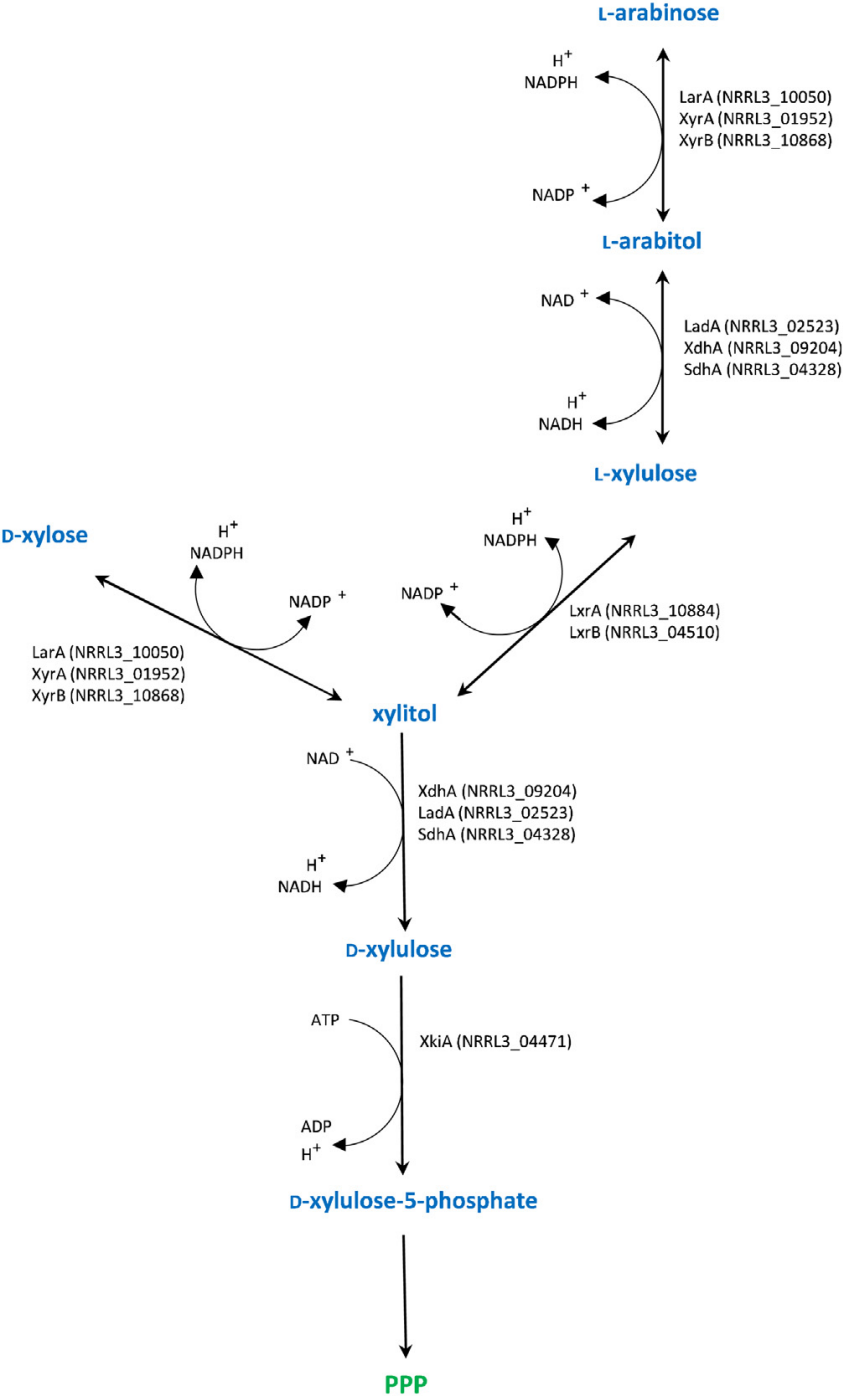
**Pentose Catabolic Pathway in *Aspergillus niger***. Enzymes involved in the pathway: LarA= l-arabinose reductase, LadA = l-arabitol dehydrogenase, LxrA, LxrB = l-xylulose reductase SdhA = sorbitol dehydrogenase, XyrA, XyrB = d-xylose reductase (the main topic of this study), XdhA = xylitol dehydrogenase, XkiA = d-xylulose kinase; PPP = Pentose Phosphate Pathway. Gene IDs are indicated in brackets. Based on Ref. [4].

*Aspergillus niger* is a saprobic fungus with a high potential for plant biomass degradation. Naturally occurring polysaccharides serve as a source of monomeric sugars that are a carbon source for the fungus [1]. Plant biomass contains cellulose, hemicelluloses and lignin. Xylan is the major hemicellulose in many plants and is composed of main chain β-1,4-linked d-xylose residues and side-groups such as l-arabinose, *p*-coumaric acid and ferulic acid. This makes d-xylose the most abundant pentose found in the nature [2,3]. d-Xylose and l-arabinose, after release from xylan, are taken up by *A. niger* and metabolized through the Pentose Catabolic Pathway (PCP). In this pathway, a number of reactions convert l-arabinose and d-xylose to d-xylulose-5-phosphate (Fig. 1).

It has been described [5] that in *A. niger* l-arabinose is reduced by l-arabinose reductase (LarA) to l-arabitol, which is then converted to l-xylulose by l-arabitol dehydrogenase (LadA). l-xylulose is reduced to xylitol by l-xylulose reductase (LxrA), while d-xylose is converted to xylitol in one step by d-xylose reductase (XyrA). Xylitol is converted in two steps to d-xylulose-5-phosphate, catalyzed by xylitol dehydrogenase (XdhA) and d-xylulose kinase (XkiA), respectively. d-Xylulose-5-phosphate is further metabolized in the pentose phosphate pathway [6,7]. However, a recent genetic study in *A. niger* revealed that multiple enzymes participate in most of the steps of the PCP [4]. Both LarA and XyrA convert L-arabinose and D-xylose to their respective polyols and this study also revealed the involvement of a second D-xylose reductase (XyrB). In this study, we have biochemically characterized XyrB, analyzed its expression profile and explored its presence in other fungi.

## 2 Materials and methods

### 2.1 Sequence analysis

Amino acid sequences were retrieved from MycoCosm (https://mycocosm.jgi.doe.gov/mycocosm/home) and the *Aspergillus* Genome database (www.aspgd.org). The amino acid sequences of *A. niger* XyrB NRRL3_10868, XyrA NRRL3_1952 [8] and LarA NRRL3_10050 [8], *Pyricularia oryzae* pentose reductase PRD1 (MGG_01404) [9] and *Aspergillus nidulans* glycerol dehydrogenase GldB AN5563 [10] were used as a query for BlastP analysis to identify the orthologs in the other fungi. Sequences were aligned using MAFFT [11] and manually corrected. Phylogenetic tree was reconstructed using MEGA6 software [12] with the Maximum Likelihood, Neighbor Joining and Minimal Evolution algorithms, using 500 bootstraps with using the glycerol dehydrogenase (GldB) group as an outgroup. The representative Maximum Likelihood tree was then displayed with bootstrap values at the nodes from all three algorithms if their support was at least 50%.

### 2.2 Expression analysis

Previously published transcriptome data [8] was used to analyze the expression of *A. niger xyrB*. Average values of expression on different sugars were compared between the wild type, and the *xlnR* and *araR* deletion strains.

### 2.3 Molecular cloning

The selected gene NRRL3_10868 without introns was codon optimized and synthesized into pET-28a(+) plasmid for production in *Escherichia coli* (Genscript Biotech, Leiden, the Netherlands). The pET-28a(+) containing synthetic gene was transformed into *E. coli* DH5α for propagation and sequencing. Then, the plasmids were isolated and transformed into *E. coli* strain BL21(DE3)pLysS (Novagen, Merck, Darmstadt, Germany) according to the manufacturer’s recommendation. The transformants were selected on Luria Bertani (LB) medium supplemented with 25 mg L^−1^ kanamycin and 17 mg L^−1^ chloramphenicol. Positive colonies were verified by colony PCR using gene-specific primers.

### 2.4 Protein production and purification

*E. coli* BL21(DE3)pLysS competent cells were transformed with plasmid pET28a(+) containing *xyrB* (NRRL3_10868). Transformed *E. coli* was grown to an OD_600_ of 0.8 in LB medium containing 34 mg L^−1^ chloramphenicol and 25 mg L^−1^ kanamycin at 37 °C with shaking 250 rpm. XyrB production was induced by addition of isopropyl β-D-1-thiogalactopyranoside (IPTG) to its final concentration of 0.1 mM and incubated overnight at 12°C with shaking 250 rpm. The cells were then harvested by centrifugation at 11,000×g for 15 min at 4°C and the cell pellet was resuspended in 30 mL of BugBuster Protein Extraction Reagent containing 2 μL Benzonase Nuclease (Merc Millipore, Darmstadt, Germany). After the incubation at 4°C with rotating mixing, the cell debris was removed by centrifugation at 11,000×g for 20 min at 4°C. Supernatant was filtered through 45 μm syringe filters (Whatman, GE Healthcare Life Sciences, Pittsburgh, PA, USA) and 25 mL of supernatant was applied to a 1 mL HisTrap FF column (GE Healthcare Life Sciences, Pittsburgh, PA, USA) at flow rate 1.0 mL min^−1^. After washing with Buffer A (20 mM HEPES, 20 mM Imidazole, 400 mM NaCl, pH 7.5) at flow rate 1.0 mL min^−1^, the bound proteins were eluted with Buffer B (20 mM HEPES, 400 mM Imidazole, 400 mM NaCl, pH 7.5) at flow rate 1.0 mL min^−1^. Fractions corresponding to absorbance peak were collected and verified by SDS-PAGE, then pooled and desalted with 20 mM HEPES pH 7.0 using an Ultracentrifugal Filter (Amicon Ultra 15 mL Filter, Merck Millipore, Darmstadt, Germany) with a MW cut-off 10 kDa. Purification parameters are listed in Supplementary file S2 (Table S2-1). Then the different dilutions of protein were again checked by SDS-PAGE. All purification steps were performed at 4°C. Protein concentration was determined using Pierce BCA Protein Assay Kit (Thermo Fisher Scientific, Waltham, MA, USA).

### 2.5 Enzymatic assays

All enzymatic assays were performed at 25°C. Reductase activities were determined in PBS buffer, pH 7.0, with 0.2 mM NADPH cofactor and 100 mM substrate. Dehydrogenase activities (reverse reactions) were performed in glycine buffer, pH 9.6, with 0.2 mM NADP and 100 mM sugar alcohol as a substrate. For the kinetic analysis of XyrB on d-xylose and l-arabinose, and d-galactose, reactions were performed in a reaction mixture of 0.2 mL total volume. The reaction mixtures contained 0.2 mM NADPH cofactor, various concentrations of d-xylose, l-arabinose and d-galactose (0−300 mM) and the appropriate amount of purified XyrB in PBS buffer, pH 7.0. The consumption of NADPH (extinction coefficient ε = 6.22 × 10^−3^ M^-1^ cm^-1^) was monitored from the reaction mixture by measuring the decrease in absorbance at 340 nm in flat-bottom microtiter plates (Grainer Bio-One, Kremsmünster, Austria) in the microplate reader (FLUOstar OPTIMA, BMG LABTECH, Ortenberg, Germany). The cofactor specificity was also tested with 0.2 mM NADH. The kinetic constants *K*_m_ and *V*_max_ were calculated from the Michaelis-Menten equation fitted to the measured data.

## 3 Results and discussion

### 3.1 Characterization of recombinant *A. niger* XyrB

The *xyrB* gene (NRRL3_10868) encodes a protein of 299 amino acids with a calculated molecular mass of 33.47 kDa and a p*I* of 6.09. It contains an aldo/keto reductase (AKR) motif between positions 22-277 (data obtained from Motif Finder https://www.genome.jp/tools-bin/search_motif_lib). The mechanism of catalysis in AKRs involves a catalytic tetrad – His, Tyr, Lys and Asp (indicated with red asterisks in Suppl. File S1) – in which the tyrosine hydroxyl group is the general acid and appears to be a proton relay from the histidine or lysine [13]. This mechanism is likely conserved in other AKRs that contain these amino acids [14]. The alignment of the amino acid sequences revealed the presence of conserved regions that are characteristic for AKRs (Suppl. File S1): the N-terminal region motif indicated with a red box, and two active region motifs indicated with green boxes (Suppl. File S1) [15]. The first region, LxxGxxxPxxGxG, is conserved in almost all sequences. Nevertheless, there are some exceptions in the glycerol dehydrogenase GCY1/YPR1 clade, and pentose reductase PRD1 clade. In *Scheffersomyces stipitis* protein ID 88249 (GCY/YPR1 clade), the first Gly in this motif (at position 15 in the sequence) is substituted by Glu, and in *Verticillium alfalfa* protein ID 801 (PRD1 clade) Leu is substituted by Thr (position 8 in the sequence) and Gly by Pro (position 11 in the sequence). In protein sequences from all clades except the GldB outgroup, a conserved Trp residue is located two residues downstream from the LxxGxxxPxxGxG motif. This residue is likely involved in selective binding and achieving specificity for d-xylose. This structure-function relationship was proposed by Kratzer et al. (2006) in the *Candida tenuis* d-xylose reductase, *Ct*XR. They showed that aldehyde-preferring AKRs at the position homologous to Trp-23 in *Ct*XR usually have a Trp residue, and the ketone-preferring AKRs at this position have mostly Tyr or Phe and in some cases other residues [16]. This pattern is also visible in our alignment (Suppl. File S1), where most of the protein sequences have the Trp residue at this position, while all sequences from the GldB outgroup and *Candida boidini* protein ID 1386 (GCY1/YPR1 clade) have a Phe residue.

The second conserved motif is GxxxxDxAxxY and it is one of the AKRs active regions. All aligned sequences contain Gly and Tyr (at the positions corresponding to 44 and 45 in *A. nidulans* AN1274). The Tyr residue is probably involved in the substrate binding as was revealed by side-directed mutagenesis of d-xylose reductase from *Debaryomyces hansenii* [17]. The GxxxxDxAxxY motif is very well conserved in all aligned proteins, even the GldB outgroup. The only exception is *Botrytis cinerea*_BC1T12462 protein ID 759 (XyrA clade) that lacks this conserved Tyr at the end of the motif. The second active region LxxxxxxxxDxxxxH contains the conserved His from the catalytic tetrad. The only differences in this motif in the aligned proteins are in the PRD1 clade, where three sequences, *Nectria haematococca* protein ID 38020, *V. alfalfa* protein ID 9347 and *P. oryzae*_MGG01404, have Asn or Val instead of Leu at the position 98. Also, in *Trichoderma reesei* protein ID 116623, Leu is substituted by Cys at this position.

All aligned sequences contain a coenzyme binding motif with some variations. Proteins from the XyrB clade have three variants of this region – IPKS, LPKS, VPKS – while the GCY1/YPR1 and unknown clades have LPKS and LAKS. Proteins from the LarA, XYR1 and PRD1 clades have only one variant – IPKS. Most of the proteins from the XyrA clade have the IPKS motif, except *T. reesei* 111218 where Ser is substituted by Thr. Proteins from the GldB outgroup have the LPKS motif, with the exception of *B. cinerea*_BC1T00765, where the conserved Lys is substituted by Met. In the *A. niger* XyrB sequence, this region consists of Ile-Pro-Lys-Ser (IPKS motif) and the conserved Arg residue situated five residues downstream (indicated with a blue asterisk in Suppl. File S1) [18]. The crystallographic studies of the *C. tenuis Ct*XR [19] and *S. stipitis* d-xylose reductase [20] showed that the Lys residue in the IPKS motif binds the 2’-phosphate group of NADPH and therefore plays a role in coenzyme binding and selectivity. The role of conserved Lys in IPKS motif was also investigated by site-directed mutagenesis. The Lys270Met mutant of *S. stipitis* d-xylose reductase showed reduced affinity for NADPH (5.1-fold lower affinity in comparison to the wild-type enzyme) while the affinity to NADH remained unchanged [21]. This residue interacts with other amino acids from the catalytic tetrad. The Lys side chain is stabilized in a position juxtaposed of the Tyr side chain through a salt-link interaction with Asp residue. The Asp side chain forms a hydrogen bond with a ribose hydroxy group of the coenzyme [22,23]. The Lys residue is conserved in all sequences except *B. cinerea*_BC1T00765, as mentioned above. As the Lys residue is considered to interact with 2’-phosphate group of NADPH, this difference may have an effect in the coenzyme preference.

**Fig. 2:**
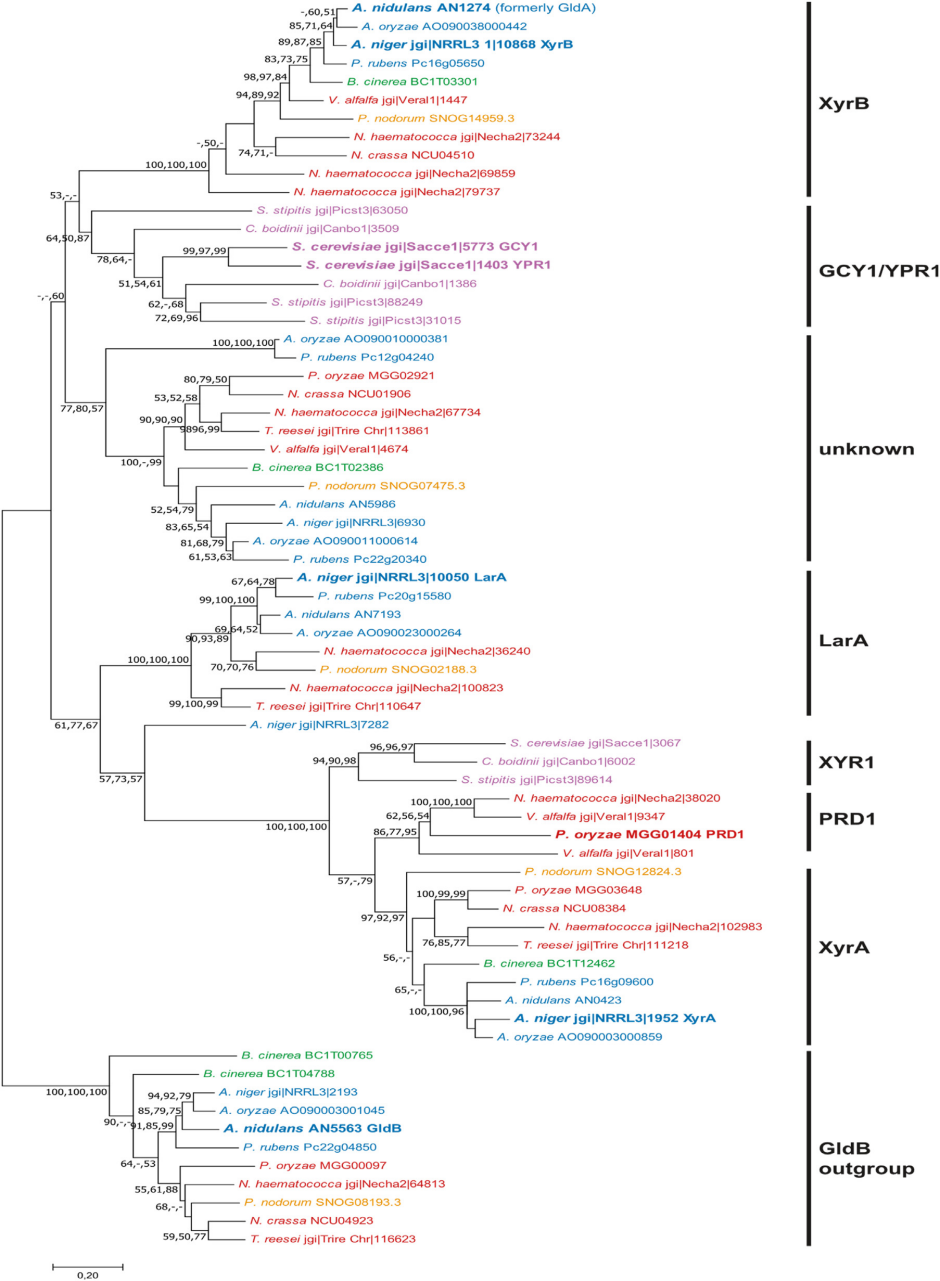
**Phylogenetic analysis of pentose reductases and related genes**. The tree is a representative Maximum Likelihood tree. Bootstrap values (50 or larger) are indicated on the nodes that are supported by the Maximum Likelihood, Neighbor Joining and Minimal Evolution algorithms in this order. XyrA/XyrB/XYR1 = d-xylose reductase, LarA = l-arabinose reductase, PRD1 = pentose reductase, GCY1/YPR1 = yeast glycerol dehydrogenases, GldB = filamentous fungal glycerol dehydrogenase. Colors reflect the taxonomic group of the fungi. Blue = Eurotiomycetes, red = Sordariomycetes, green = Leotiomycetes, orange = Dothidiomycetes, pink = Saccharomycetes. Amino acid sequences used as a query in a BlastP search are in larger font and boldface.

Previous studies into d-xylose reductases mainly focused on species from three fungal clades: Eurotiomycetes, Sordariomycetes and Saccharomycetes. We therefore focused in particular on these in our phylogenetic analysis, including four Eurotiomycetes (*A. niger*, *A. nidulans*, *Aspergillus oryzae*, *Penicillium rubens*), five Sordariomycetes (*V. alfalfa*, *N. haematococca*, *Neurospora crassa*, *P. oryzae*, *T. reesei*) and three Saccharomycetes (*Saccharomyces cerevisiae*, *S. stipitis*, *Candida boidinii*) species, but also adding one Dothidiomycete (*Phaeosphaeria nodorum*) and one Leotiomycete (*B. cinerea*) species. Orthologs of *A. niger xyrA* [24], *xyrB* (this study) and *larA* [24], *P. oryzae PRD1* [9] and *A. nidulans gldB* [10] were obtained from these fungal genomes using a BLASTP search on the AspGD (www.aspgd.org) [25] and MycoCosm (https://mycocosm.jgi.doe.gov/mycocosm/home) [26] databases. The best hits for these queries were extracted and used for construction of the phylogenetic tree (Fig. 2). The different enzymes separated as clear branches with good bootstrap support. The XyrB clade and the yeast glycerol dehydrogenase (GCY1/YPR1) clade separate from a common node, and are adjacent to a clade of unknown function consisting of one or two enzymes from each tested filamentous fungus. The LarA clade separates from the XyrA/PRD1/XYR1 clade. Most tested fungi have a single member in this last clade, except for those species containing one or two PRD1 enzymes (Fig. 2). The Saccharomycete species only contain a single XYR1 enzyme and two or three GCY1/YPR1 enzymes, but do not have members of any of the other clades, including the filamentous fungal glycerol dehydrogenase (GldB) branch. This branch contains a single member of each of the filamentous fungi included in the analysis, except for *B. cinerea* (2 members) and *V. alfalfa* (0 members). A similar result was observed for the XyrB clade with the exception of *N. haematococca* (3 members) and *P. oryzae* (0 members). The presence of LarA was less conserved, as it is absent in three of the Sordariomycetes species and in *B. cinerea*. In contrast, *N. haematococca* and *P. nodorum* contain two paralogs each in this clade.

Although *A. niger* XyrB is d-xylose reductase, it is distant from the other d-xylose reductase clade. Moreover, it lacks the yeast d-xylose reductase coenzyme binding motif (GxxxGxG(D/E)) [15].

### 3.2 Expression analysis of *xyrB*

**Fig. 3:**
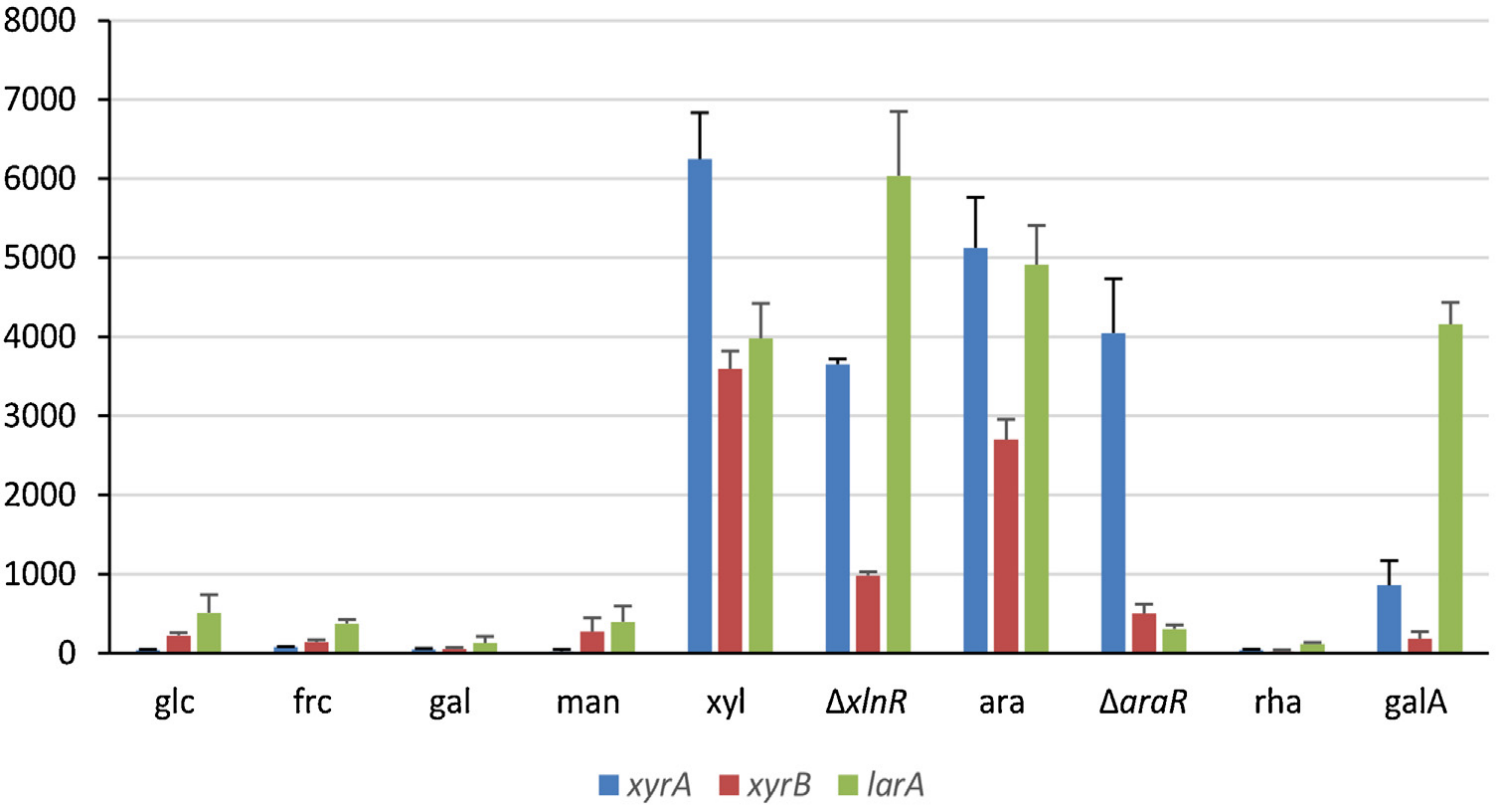
**Expression profiles for d-xylose reductase *xyrA*, d-xylose reductase *xyrB* and l-arabinose reductase *larA* from *A. niger***. Expression values of the genes (based on normalized probe intensities) were obtained from previously published transcriptome (microarray) study [8]. If only a carbon source is mentioned, than this is the expression value of a wild type strain transferred for 2 h to that carbon source. Glc = d-glucose, frc = d-fructose, gal = d-galactose, man = d-mannose, xyl = d-xylose, ara = d-arabinose, rha = l-rhamnose, galA = d-galacturonic acid. Δ*xlnR* = *xlnR* deletion strain transferred for 2 h to d-xylose, Δ*araR* = *araR* deletion transferred for 2 h to l-arabinose. The error bars represent standard deviation of biological duplicates.

Previously it was shown that *xyrA* is under control of the xylanolytic regulator XlnR [3], while *larA* is under control of the arabinanolytic regulator AraR [27] in *A. niger*. Expression of *xyrB* was compared to *xyrA* and *larA* in previously published transcriptome data of *A. niger* [8], and induction of expression on both d-xylose and l-arabinose, with a higher expression level on d-xylose, was observed (Fig. 3). The expression levels after 2 h on d-xylose and l-arabinose are >15-fold and 12-fold higher, respectively, compared to d-glucose. Expression of *xyrB* on d-xylose was lower than that of *xyrA* and comparable to *larA*, while its expression on l-arabinose was lower than that of both *xyrA* and *larA*. The induction of *xyrB* expression by both d-xylose and l-arabinose is typical for fungal d-xylose and l-arabinose reductases encoding genes [3,28,29]. Expression of *A. niger xyrB* was strongly reduced in the *xlnR* deletion strain on d-xylose and in the *araR* deletion strain on l-arabinose, indicating control of its expression by both regulators. This correlates with the presence of binding sites for XlnR (GGCTAR) [30] and AraR (CGGDTAAW) [31] in its promoter.

### 3.3 XyrB production and characterization

*A. niger* XyrB was recombinantly produced in *E. coli* and purified. Fractions that corresponded to absorbance peak in the FPLC were verified by SDS-PAGE (Suppl. File S2). Fractions 3–8 consisted recombinant protein and were pooled and desalted. The protein dilutions were again checked by SDS-PAGE (Suppl. File S2).

Specific activity analysis of *A. niger* XyrB shows that the enzyme is able to convert a wide range of sugars and polyols (Table 2). The range of substrates converted by XyrB is comparable to the pentose reductase PRD1 from *P. oryzae* [9]. XyrB shows the highest specific activity towards d-galactose, d-xylose and l-arabinose, 136 U mg^−1^, 83.9 U mg^−1^ and 98.5 U mg^-1^, respectively (Table 2). The *A. nidulans* XyrB ortholog (AN1274, previously named GldA) represents a comparable substrate specificity profile towards d-xylose and l-arabinose, with specific activity of 108.8 U mg^−1^ and 134.3 U mg^−1^, respectively (de Vries, unpublished data). Interestingly, *A. niger* d-xylose reductase XyrA has no activity with d-galactose [24]. d-Galactose has the same configuration on C4 as l-arabinose (C-4(L) configuration). Therefore, as d-galactose is a mimic of l-arabinose, it is not surprising that it can also be converted by XyrB, but it cannot serve as an evidence for biological function of XyrB. Values of XyrB specific activity on d-xylose and l-arabinose are similar, while the specific activity towards l-arabinose is slightly higher.

There was no activity detected with NADH as a cofactor, which also is typical for other fungal D-xylose reductases [13,32,33]. Only a few D-xylose reductases have been reported to be active in the presence of both NADH and NADPH as a cofactor, such as those from *N. crassa* [34], *C. tenuis* [35,36] and from the thermophilic fungus C*haetomium thermophilum* [37,38]. However, also for these enzymes the activity with NADPH was higher than with NADH, for *N. crassa* and *C. thermophilum* even 11-fold.

**Fig. 4:**
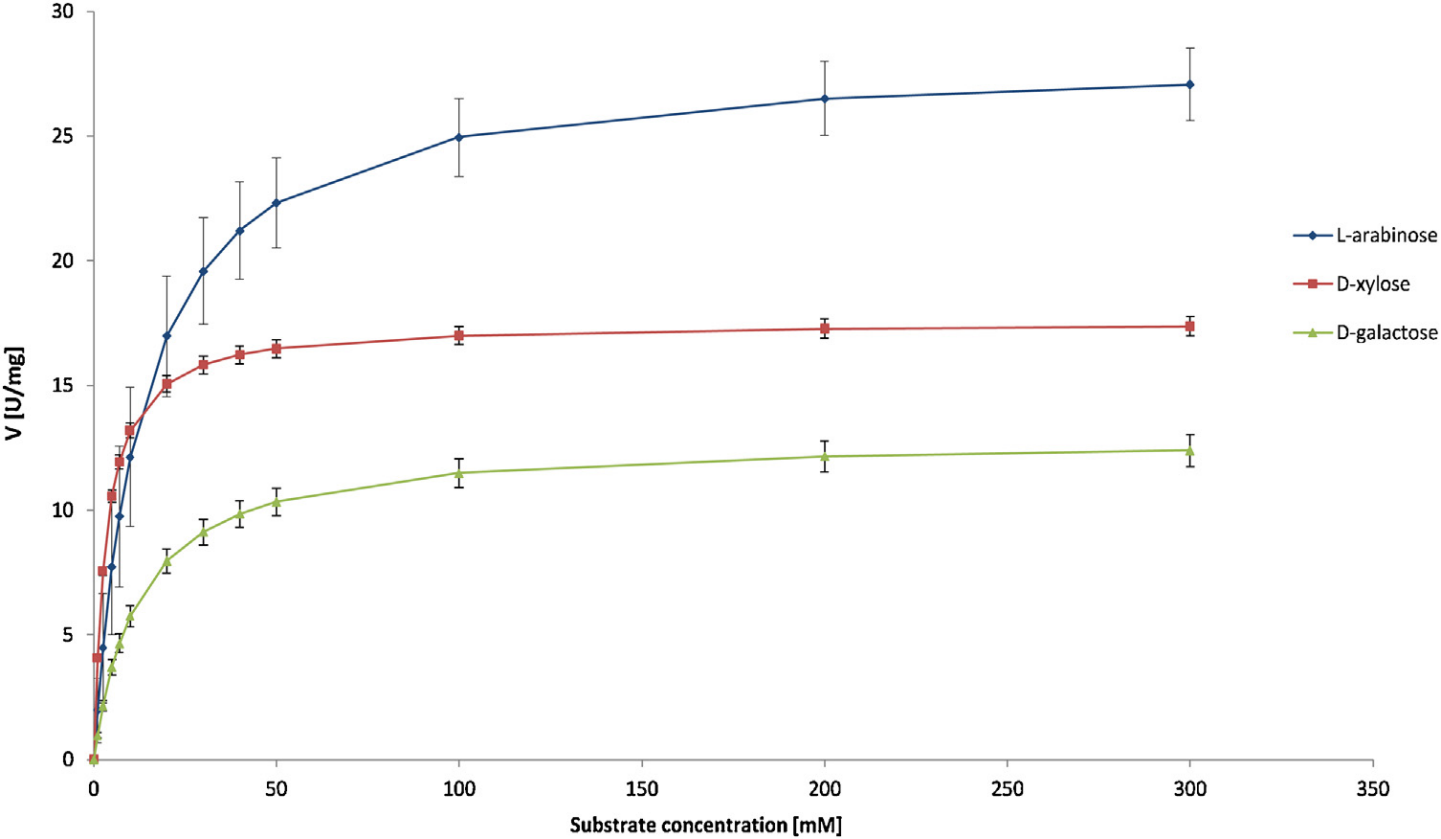
**Kinetic characterization of XyrB with different substrates**: d-xylose, l-arabinose and d-galactose. The Michaelis-Menten constants were estimated from fitting the Michaelis-Menten kinetics to the data points. Vertical bars represent standard deviation from three assay repeats.

Kinetic analysis of XyrB was performed with three substrates: d-xylose, l-arabinose and d-galactose (Fig. 4). Based on its substrate specificity (Table 1), XyrB could be involved in d-galactose conversion in *A. niger*. However, *xyrB* is not expressed in the presence of d-galactose, which argues against a role for XyrB in the d-galactose oxidoreductive pathway [39]. Moreover, the results from kinetic analysis support this conclusion. The *K*_m_ and *V*_max_ values for d-xylose and l-arabinose are 3.3 mM and 9.9 mM, and 17.6 U mg^−1^ and 19.9 U mg^−1^, respectively. For d-galactose these values are: *K*_m_ =12.5 mM and *V*_max_ = 12.9 U mg^−1^. Therefore, XyrB from *A. niger* has 3-fold higher affinity for d-xylose than for l-arabinose and nearly 4-fold higher than for d-galactose. The enzyme turnover (*k*_cat_) for d-xylose is 587.8 min^−1^, for l-arabinose 666.9 min^−1^ and for d-galactose 432.1 min^−1^. The catalytic efficiency (*k*_cat_/*K*_m_) of XyrB is 177.7 mM^−1^ min^−1^, 67.2 mM^−1^ min^−1^ and 34.6 mM^−1^ min^−1^ for d-xylose, l-arabinose and d-galactose, respectively. Results of the kinetic analysis of XyrB show that, although the rates of conversion of l-arabinose into L-arabitol and d-xylose into xylitol are similar, the values of substrate specificity indicate that the preferred substrate for XyrB is d-xylose.

**Table 1:** Comparison of substrate specificity of *Aspergillus niger* XyrB and *Aspergillus nidulans* AN1274. Values are the average of triplicate assays and the standard deviation is given behind the values.

	Substrate	XyrB specific activity [U mg^−1^]	AN1274 specific activity [U mg^−1^]
**Reductase activity**	d-xylose	**83.9 ± 11.2**	**108.8 ± 13.7**
	l-arabinose	**98.5 ± 4**	**134.3 ± 9.6**
	d-glucose	16.7 ± 4.6	12.5 ± 1.9
	d-mannose	9.2 ± 3	N.T.
	d-fructose	13.4 ± 5.9	4.3 ± 1.2
	l-rhamnose	11.7 ± 6.6	N.T.
	d-galactose	**136.0 ± 4.6**	**N.T.**
	l-sorbose	32.2 ± 0.5	N.T.
	d-ribose	35.0 ± 4.0	40.0 ± 4.3
	d-arabinose	15.1 ± 4.8	0.9 ± 0.2
	l-xylose	9.6 ± 0	N.T.
**Dehydrogenase activity**	xylitol	**11.3 ± 0.8**	**2.5 ± 0.4**
	l-arabitol	**9.2 ± 0.5**	**2.0 ± 0.7**
	ribitol	N.D.	N.T.
	galactitol	**9.8 ± 0.5**	**N.T.**
	d-arabitol	N.D.	N.D.
	sorbitol	N.D.	N.T.
	mannitol	N.D.	4.9 ± 0.6
	glycerol	**3.8 ± 0.7**	**4.2 ± 0.5**

N.T. = not tested, N.D. = not detected.

The kinetic properties of XyrB were compared to those of other published fungal pentose reductases. While the affinity for d-xylose and l-arabinose is in a similar range for XyrB and XyrA, the *K*_m_ for both substrates is the lowest for XyrB (Table 2). Comparing the enzyme turnover for d-xylose among other fungal d-xylose reductases XyrB and CtXR have the lowest turnover – 587.8 min^−1^ and 552 min^−1^ respectively. For L-arabinose the highest enzyme turnover was observed for d-xylose reductase from *S. stipitis*. But XyrBt has the highest catalytic efficiency for both d-xylose and l-arabinose (Table 2).

**Table 2:** Comparison of kinetic constants of NADPH dependent d-xylose reductases and other pentose reductases from different fungal species.

		d-xylose				l-arabinose				Ref.
		*K*_m_ [mM]	*V*_max_ [U mg^-1^]	*k_cat_* [min^−1^]	*k_cat_/K_m_* [mM^−1^ min^−1^]	*K*_m_ [mM]	*V*_max_ [U mg^-1^]	*k_cat_* [min^−1^]	*k_cat_/K_m_* [mM^−1^ min^−1^]	
Fungal d-xylose reductases	*Aspergillus niger* XyrB	**3.3**	**17.6**	**587.8**	**177.7**	**9.9**	**19.9**	**936.4**	**71.1**	This study
	*Aspergillus niger* XyrA^a^	6	20.5	740.1	123.4	21	20	722	34.4	[24]
	*Rhizomucor pusillus* RpXR	28.1	34.8	1280	45.6	41.7	30.1	1101	26.4	[2]
	*Neurospora crassa* XR	34.4	93.5	3600	110	40	46.8	1800	45	[34]
	*Chaetomium thermophilum Ct*XR	22.3	14.1	552	26	62.5	13.5	528	8.4	[37]
	*Trichoderma reesei*	25	26.9	985.8	39.2	41.6	20.9	765.6	18.8	[40]
	*Candida tropicalis*	30	50.7	2940	99	N.M.	N.M.	N.M.	N.M.	[41]
	*Candida intermedia*	82	24.3	876	10.68	N.M.	N.M.	N.M.	N.M.	[33]
	*Scheffersomyces stipitis*	42	23.2	1508.5	35.92	40	34.5	2243.2	56.1	[32]
	*Saccharomyces cerevisiae*	13.6	23.4	864	63.6	N.M.	N.M.	N.M.	N.M.	[42]
Other fungal pentose reductases	*Aspergillus niger* LarA	155	1.65	59.42	0.38	54	2.35	84.62	1.57	[24]
	*Pyricularia oryzae* PRD1	20	0.24	9.05	0.46	20	0.36	13.36	0.68	[9]

N.M. = not mentioned.

a Results for unpurified enzyme.

## 4 Conclusions

In this study, we combined data obtained from transcriptome, phylogenetic and biochemical analysis to reveal the role of XyrB in *A. niger* carbon metabolism. The expression analysis of *xyrB* confirmed the results obtained from specific activity analysis. XyrB has activity towards a wide range of substrates, but with strongly varying levels. Although XyrB is able to convert of d-glucose, l-rhamnose, d-mannose and d-fructose, the specific activity is low and there was no expression of the *xyrB* gene observed in the presence of these sugars. The high expression of *xyrB* on d-xylose and l-arabinose and regulation by both XlnR and AraR suggested that XyrB is involved in the conversion of both pentoses. Genetic analysis of the role of XyrA, XyrB and LarA revealed that all three enzymes contribute to D-xylose and L-arabinose conversion, but not in equal amounts. Together with the differences in regulation of the expression of the corresponding genes, this likely provides *A. niger* with the tools to modify the relative l-arabinose and d-xylose reductase activity both at the expression and specificity level, in response to the abundance of these two pentoses. The differences between these enzymes are particularly relevant when using them in biotechnological processes, such as the production of xylitol.

## Declaration of Competing Interest

The authors declare that they have no known competing financial interests or personal relationships that could have appeared to influence the work reported in this paper.
