# Supplementary material for: Characterization of d-xylose reductase, XyrB, from Aspergillus niger
Source: Biotechnol Rep (Amst). 2021 Mar 15;30:e00610. doi: 10.1016/j.btre.2021.e00610 (PMC8020424; doi:10.1016/j.btre.2021.e00610)
Supplement: Supplementary file 1 [file mmc1.pdf]

Supplementary File 1 S1

```
                                             10        20        30        40        50
                                    ....|....|....|....|....|....|....|....|....|....|
A.nidulans_AN1274                   -------------------------------------------------- 1
A.oryzae_AO090038000442             -------------------------------------------------- 1
A.niger|NRRL3|10868_XyrB            -------------------------------------------------- 1
P.rubens_Pc16g05650                 -------------------------------------------------- 1
B.cinerea_BC1T03301                 -------------------------------------------------- 1
V.alfalfa|Veral1|1447               -------------------------------------------------- 1
P.nodorum_SNOG_14959.3              -------------------------------------------------- 1
N.haematococca|Necha2|73244         -------------------------------------------------- 1
N.crassa_NCU04510                   --------------------------------MLP-VSRAIPR--IPNIT 15
N.haematococca|Necha2|69859         -------------------------------------------------- 1
N.haematococca|Necha2|79737         -------------------------------------------------- 1
S.stipitis|Picst3|63050             -------------------------------------------------- 1
C.boidini|Canbo1|3509               -------------------------------------------------- 1
S.cerevisiae|Sacce1|5773_GCY1       -------------------------------------------------- 1
S.cerevisiae|Sacce1|1403_YPR1       -------------------------------------------------- 1
C.boidini|Canbo1|1386               -------------------------------------------------- 1
S.stipitis|Picst3|88249             -------------------------------------------------- 1
S.stipitis|Picst3|31015             -------------------------------------------------- 1
A.oryzae_AO090010000381             -------------------------------------------------- 1
P.rubens_Pc12g04240                 -------------------------------------------------- 1
P.oryzae_MGG_02921                  -------------------------------------------------- 1
N.crassa_NCU01906                   -------------------------------------------------- 1
N.haematococca|Necha2|67734         -------------------------------------------------- 1
T.reesei|Trire_Chr|113861           -------------------------------------------------- 1
V.alfalfa|Veral1|4674               -------------------------------------------------- 1
B.cinerea_BC1T02386                 -------------------------------------------------- 1
P.nodorum_SNOG07475.3               -------------------------------------------------M 1
A.nidulans_AN5986                   -------------------------------------------------- 1
A.oryzae_AO090011000614             -------------------------------------------------- 1
P.rubens_Pc22g20340                 -------------------------------------------------- 1
A.niger|NRRL3|6930                  -------------------------------------------------- 1
A.niger|NRRL3|10050_LarA            -------------------------------------------------- 1
P.rubens_Pc20g15580                 -------------------------------------------------- 1
A.nidulans_AN7193                   -------------------------------------------------- 1
A.oryzae_AO090023000264             -------------------------------------------------- 1
N.haematococca|Necha2|36240         -------------------------------------------------- 1
P.nodorum_SNOG_02188.3              -------------------------------------------------- 1
N.haematococca|Necha2|100823        -------------------------------------------------- 1
T.reesei|Trire_Chr|110647           -------------------------------------------------- 1
A.niger|NRRL3|7282                  -------------------------------------------------- 1
S.cerevisiae|Sacce1|3067            -------------------------------------------------- 1
C.boidini|Canbo1|6002               -------------------------------------------------- 1
S.stipitis|Picst3|89614             -------------------------------------------------- 1
N.haematococca|Necha2|38020         -------------------------------------------------- 1
V.alfalfa|Veral1|9347               -------------------------------------------------- 1
P.oryzae_MGG01404                   -------------------------------------------------- 1
V.alfalfa_|Veral1|801               -------------------------------------------------- 1
P.nodorum_SNOG_12824.3              -MVAGRFCRTSINTVRSFTTAVVPRSSFFPPVRTCISRTKAPSFRPT--Y 47
P.oryzae_MGG03648                   -------------------------------------------------- 1
N.crassa_NCU08384                   -------------------------------------------------- 1
N.haematococca|Necha2|102983        -------------------------------------------------- 1
T.reesei|Trire_Chr|111218           -------------------------------------------------- 1
B.cinerea_BC1T12462                 -------------------------------------------------- 1
P.rubens_Pc16g09600                 -------------------------------------------------- 1
A.nidulans_AN0423                   -------------------------------------------------- 1
A.niger|NRRL3|1952_XyrA             -------------------------------------------------- 1
A.oryzae_AO090003000859             -------------------------------------------------- 1
B.cierea_BC1T00765                  -------------------------------------------------- 1
B.cinerea_BC1T04788                 -------------------------------------------------- 1
```

```
A.niger|NRRL3|2193             -------------------------------------------------- 1
A.oryzae_AO090003001045        -------------------------------------------------- 1
A.nidulans_AN5563              -------------------------------------------------- 1
P.rubens_Pc22g04850            -------------------------------------------------- 1
P.oryzae_MGG00097              -------------------------------------------------- 1
N.haematococca|Necha2|64813    -------------------------------------------------- 1
P.nodorum_SNOG08193.3          -------------------------------------------------- 1
N.crassa_NCU04923              -------------------------------------------------- 1
T.reesei|Trire_Chr|116623      -------------------------------------------------- 1
Clustal Consensus

                                        60        70        80        90       100
                               ....|....|....|....|....|....|....|....|....|....|
A.nidulans_AN1274              --------MS-LEHT-------------------KKVYTLNTGDKIPAIG 22
A.oryzae_AO090038000442        --------MASLQHT-------------------KKTYTLNTGDKIPAIG 23
A.niger|NRRL3|10868_XyrB       --------MSNLEHT-------------------KKVYTLNTGDKIPAVG 23
P.rubens_Pc16g05650            --------MS-NQHT-------------------QKTYTLNTGAKIPAIG 22
B.cinerea_BC1T03301            --------MSANNVT-------------------SKTFTLNTGAKIPAIG 23
V.alfalfa|Veral1|1447          --------MSGTTQT-------------------SRTFTLNTGDKLPAVG 23
P.nodorum_SNOG_14959.3         --------MTPKNWT-------------------QESFTLNTGAKIPAIG 23
N.haematococca|Necha2|73244    --------MTYQENT-------------------TKKYVLNTGDSMPALG 23
N.crassa_NCU04510              NTARYFFSKTNTMAPL------------------SKTHKLNTGDEIPAVG 47
N.haematococca|Necha2|69859    -----------MYP--------------------SRTFILNTGDEIPAVG 19
N.haematococca|Necha2|79737    --------MDAKNAT-------------------SLRYKLNTGASIPAIG 23
S.stipitis|Picst3|63050        ------MVALTND---------------------SVVYTLNNGVKIPAIG 23
C.boidini|Canbo1|3509          ------MSTAAKS---------------------TSILRLNTGVSIPQIG 23
S.cerevisiae|Sacce1|5773_GCY1  ------MPATLHDS--------------------TKILSLNTGAQIPQIG 24
S.cerevisiae|Sacce1|1403_YPR1  ------MPATLKNS--------------------SATLKLNTGASIPVLG 24
C.boidini|Canbo1|1386          ------MSPEIC----------------------TKKLKLNSGYEIPNVG 22
S.stipitis|Picst3|88249        -------MPATKA---------------------TKVFQLNDETSIPGVG 22
S.stipitis|Picst3|31015        ------MATSYKS---------------------SKVYKLNSGETIPALG 23
A.oryzae_AO090010000381        --------MSP-----------------------PSHFKLNTGAQIPAVG 19
P.rubens_Pc12g04240            --------MAP-----------------------STHFKLNTGAQIPTVG 19
P.oryzae_MGG_02921             --------MA------------------------DKDFTLNTGAKIPAFG 18
N.crassa_NCU01906              --------MA------------------------TKTFKLNTGANIPALG 18
N.haematococca|Necha2|67734    --------MA------------------------PTSFKLNNGQEIPAVG 18
T.reesei|Trire_Chr|113861      --------MV------------------------ATSFKLNNGLEIPAVG 18
V.alfalfa|Veral1|4674          --------MA------------------------DQTFKLNTGASIPAIG 18
B.cinerea_BC1T02386            --------MA------------------------DTRFKLNTGAEIPALG 18
P.nodorum_SNOG07475.3          NPALSKFILKA-TPD---------SILNPSPDM-NTAIPLNTGATIPALG 40
A.nidulans_AN5986              ------MSLA------------------------DTTYKLNTGAEIPALG 20
A.oryzae_AO090011000614        ------MAVA------------------------DTRFKLNTGAEIPALG 20
P.rubens_Pc22g20340            ------MAAC------------------------DTRFKLNTGAEIPALG 20
A.niger|NRRL3|6930             --MGSTSTVA------------------------DTRFKLNTGAEIPALG 24
A.niger|NRRL3|10050_LarA       --------MSL-----------------------GKKVTLNSGAQIPQLG 19
P.rubens_Pc20g15580            --------MSL-----------------------GKKVTLNTGHQIPQLG 19
A.nidulans_AN7193              --------MSL-----------------------GKKVTLNTGAQIPQLG 19
A.oryzae_AO090023000264        --------MSL-----------------------GKKVTLNSGAEIPTLG 19
N.haematococca|Necha2|36240    --------MSL-----------------------GRTVLLNSGHKIPQLG 19
P.nodorum_SNOG_02188.3         --------MSL-----------------------GRTVKLNTGASIPQLG 19
N.haematococca|Necha2|100823   --------MSF-----------------------GRTVTLNSGHKVPVIG 19
T.reesei|Trire_Chr|110647      --------MSS-----------------------GRTVTLNTGYKIPQIG 19
A.niger|NRRL3|7282             --------MAL-----------------------NRTFKLNTGYDMPAVG 19
S.cerevisiae|Sacce1|3067       --------MSS-------------------------LVTLNNGLKMPLVG 17
C.boidini|Canbo1|6002          --------MSS------------------------ALLTLNNGLKMPQIG 18
S.stipitis|Picst3|89614        --------MPS--------------------------IKLNSGYDMPAVG 16
N.haematococca|Necha2|38020    --------MAP------------------------STIKLASGHEMPLVG 18
V.alfalfa|Veral1|9347          --------MGK------------------------STITLASGHEMPLVG 18
P.oryzae_MGG01404              --------MAK------------------------TSIKLNSGYEMPLVG 18
V.alfalfa_|Veral1|801          --------MLT------------------------KSLLTSFPKYIPQVG 18
P.nodorum_SNOG_12824.3         SNRNFFATMAVNT----------------------PYITLNDGNKMPQVG 75
```

```
P.oryzae_MGG03648              --------MAS------------------------PTMKLNNGLDMPQVG 18
N.crassa_NCU08384              --------MV-------------------------PAIKLNSGFDMPQVG 17
N.haematococca|Necha2|102983   --------MAD-I----------------------PTVKLSSGHEMPQVG 19
T.reesei|Trire_Chr|111218      --------MAS------------------------PTLKLNSGYDMPQVG 18
B.cinerea_BC1T12462            --------MAS------------------------PTLKLNSGHEMPQVG 18
P.rubens_Pc16g09600            --------MVA------------------------PTVKLSSGYEMPLVG 18
A.nidulans_AN0423              --------MSP------------------------PTVKLNSGYDMPLVG 18
A.niger|NRRL3|1952_XyrA        --------MAS------------------------PTVKLNSGYDMPLVG 18
A.oryzae_AO090003000859        --------MAS------------------------PTVKLNSGHDMPLVG 18
B.cierea_BC1T00765             --------MT------------------------IPTLKLNTGAPIPALG 18
B.cinerea_BC1T04788            ---MAAPAVT------------------------GPLIKLNNGVQMPAFG 23
A.niger|NRRL3|2193             --------MSS-----------------------GQTFTLSNGVKIPGVG 19
A.oryzae_AO090003001045        --------MSN-----------------------GKTFTLSNGVKIPGVG 19
A.nidulans_AN5563              --------MSS-----------------------GKTFKLSNGVTIPAVG 19
P.rubens_Pc22g04850            --------MSS-----------------------GKTFTLSNGVTIPAVG 19
P.oryzae_MGG00097              ----MPATLA------------------------NKTFKLNNGVEIPAVG 22
N.haematococca|Necha2|64813    ------MTSN------------------------TTTFTLNNGVKIPGLG 20
P.nodorum_SNOG08193.3          -----MAPLAN-----------------------QKTYKLNNGVEIPAVG 22
N.crassa_NCU04923              ---MTGFTAA------------------------NTTYTLNNGVRIPAVG 23
T.reesei|Trire_Chr|116623      --------MA------------------------SKTYTLNTGAKIPAVG 18
Clustal Consensus                                                   :* .*

                                        110       120       130       140       150
                               ....|....|....|....|....|....|....|....|....|....|
A.nidulans_AN1274              L---GTWQSKP--NEVREAVKNALLKGYRHIDTALAYGNEAEVGQGIKD- 66
A.oryzae_AO090038000442        L---GTWQSKP--NEVREAVKNALLKGYRHIDTALAYGNEAEVGQGIKD- 67
A.niger|NRRL3|10868_XyrB       L---GTWQSKP--NEVREAVKNALLKGYRHIDTALAYGNEAEVGQGIKD- 67
P.rubens_Pc16g05650            L---GTWQSKP--NEVRVAVRDALLAGYRHIDTALAYGNEAEVGAGIKD- 66
B.cinerea_BC1T03301            L---GTWQSPP--GEVEKAVEIALRKGYRHIDTALAYGNESEVGQGIKA- 67
V.alfalfa|Veral1|1447          L---GTWQSKP--NEVKHAVEAALRQGYRHIDTALAYGNEREVGDGIKA- 67
P.nodorum_SNOG_14959.3         L---GTWQSKP--GEVREAVKAAIQAGYRHIDTALAYGNEKEVGEGIKD- 67
N.haematococca|Necha2|73244    L---GTWQSKP--GEVELAVQWALECGYRHIDTAFAYGNEKEVGAGIRA- 67
N.crassa_NCU04510              L---GTWQSKP--GQVEKAVEAALRAGYTHIDTAYAYGNEKEVGQGIKA- 91
N.haematococca|Necha2|69859    L---GTWRSNP--RQVSTAVETALRAGYRHIDTALAYGNEQEVGNGIRA- 63
N.haematococca|Necha2|79737    L---GTWQSAE--DETRDAVKYALQNGYRHIDTAFNYKNEKEVGDGIRA- 67
S.stipitis|Picst3|63050        L---GTWQASA-PGHASNATKVALQNGYRHIDTAAIYKNEEEVGQGLKD- 68
C.boidini|Canbo1|3509          L---GTWQSTE--EEVYGAVLAAIKAGYKHIDTAAIYKNETAVGRAIKD- 67
S.cerevisiae|Sacce1|5773_GCY1  L---GTWQSKE--NDAYKAVLTALKDGYRHIDTAAIYRNEDQVGQAIKD- 68
S.cerevisiae|Sacce1|1403_YPR1  F---GTWRSVD--NNGYHSVIAALKAGYRHIDAAAIYLNEEEVGRAIKD- 68
C.boidini|Canbo1|1386          F---GTFRASE--EDTYNSVLTALKNGYKHIDTAYVYRNESSVGKAIKD- 66
S.stipitis|Picst3|88249        L---GTWQSTN--DEVYNAVLTALRYGYRHIDTAAAYGNESVIGRAIKD- 66
S.stipitis|Picst3|31015        L---GTWQALA--DDVYKAVLFALKTGYRHIDSALAYGNEEPVGRAIRD- 67
A.oryzae_AO090010000381        L---GTWRSEP--GQVRQAVSYALKNGYTHIDAALIYGNEHEVGQGIKD- 63
P.rubens_Pc12g04240            L---GTWRSEP--GQVRQAVSFALKNGYSHIDAALIYGNEHEVGQGIKD- 63
P.oryzae_MGG_02921             L---GTWQGDK--GVIKEAVLTAIKSGYRLIDGAYVYGNEEEVGQGIREA 63
N.crassa_NCU01906              L---GTWQGES--TQVKDAVVAALKSGYRLIDTAYCYGNEEHVGAGLKEA 63
N.haematococca|Necha2|67734    L---GTWQSAP--GQVEAAVAFALKDGYKMVDCAYCYGNEEEVGAGLKAA 63
T.reesei|Trire_Chr|113861      L---GTWQSKA--GEVKAAVSYALQIGYKLIDGAYCYGNEDEVGEGLKEA 63
V.alfalfa|Veral1|4674          L---GTWQGEA--GKVKAAVSFAVQNGYKLVDCAYCYANEDEVGEGLKDA 63
B.cinerea_BC1T02386            L---GTWQSEP--GAVAKAVAYALSVGYKHIDCAYVYGNEEEVGQGLKEA 63
P.nodorum_SNOG07475.3          L---GTWQSAP--GEVKKAVVHAIESGYRHIDCAFCYQNEDEVGEALQDV 85
A.nidulans_AN5986              L---GTWQSAP--GEVSAAVYHALKVGYRHIDAAQCYGNETEVGEGIKRA 65
A.oryzae_AO090011000614        L---GTWQSQP--GEVEKAVSHAISVGYRHIDGAFCYQNEEEVGKGIRDA 65
P.rubens_Pc22g20340            L---GTWQSQP--GEVARAVSHAIKVGYRHIDAALCYGNENEVGQGIKEA 65
A.niger|NRRL3|6930             L---GTWQSGP--GEVEKAVAHAISVGYRHIDTAFAYGNEGEVGKGIKAA 69
A.niger|NRRL3|10050_LarA       F---GTWQSAP--GQVGDAVYEALKAGYRHLDLATIYQNQREVAEGIKRA 64
P.rubens_Pc20g15580            F---GTWQSAP--GQVGEAVYEALKAGYRHLDLATIYQNQREVAEGIKRA 64
A.nidulans_AN7193              F---GTWQSAP--GQVGEAVYQALKVGYRHLDLATIYQNQREVAEGIKRA 64
A.oryzae_AO090023000264        F---GTWQSAP--GEVGEAVYQALKAGYRHLDLATIYQNQKEIAVGIKRA 64
N.haematococca|Necha2|36240    Y---GTWQSSP--GEVALGVFEALKVGYRHLDLAKVYGNQPEVAEGLKRA 64
P.nodorum_SNOG_02188.3         F---GTWQAEP--GQVKVAVLEALKAGYRHLDLAKVYGNQKEIAEALKEA 64
```

```
                                                                  *    *
N.haematococca|Necha2|100823       Y---GTWQSAP--GEVGAGVYEALKAGYRHLDLAKIYQNQREVGEGLKKA 64
T.reesei|Trire_Chr|110647          Y---GTWQAAP--GEVGAGVFEALKVGYRHLDLAKVYGNQKEVGEGIKKA 64
A.niger|NRRL3|7282                 L---GTWQSKK--DEVRDAVIAALKCGYRHIDAAAVYGNEQEVGDGMRL- 63
S.cerevisiae|Sacce1|3067           L---GCWKIDK--KVCANQIYEAIKLGYRLFDGACDYGNEKEVGEGIRKA 62
C.boidini|Canbo1|6002              F---GCWKVDN--ATCAETIYEAIKVGYRLFDGAMDYGNEKEVGEGINKA 63
S.stipitis|Picst3|89614            F---GCWKVDV--DTCSEQIYRAIKTGYRLFDGAEDYANEKLVGAGVKKA 61
N.haematococca|Necha2|38020        F---GLWKVPA--DQAAETVYNAIKAGYRLFDGAYDYQNEKEAGEGIKRA 63
V.alfalfa|Veral1|9347              F---GLWKVPR--ETCADTVYNAIKVGYRLFDGAYDYQNEKEAGEGIQRA 63
P.oryzae_MGG01404                  F---GIWKVPV--DKTAQAVYDAIKLGYRQIDGAYDYTNSKEAGEGVRRA 63
V.alfalfa_|Veral1|801              F---GLWKVPA--AQTADAVYGAIKAGYRHIDGAYGYANSAEAGQGVRRA 63
P.nodorum_SNOG_12824.3             F---GLWKVDN--ATCADTVYNAIKTGYRLFDGACDYGNEVECGQGVARA 120
P.oryzae_MGG03648                  F---GLWKVEN--SVCADVVYNAIKAGYRLFDGACDYGNEVECGQGVKRA 63
N.crassa_NCU08384                  F---GLWKVDG--SIASDVVYNAIKAGYRLFDGACDYGNEVECGQGVARA 62
N.haematococca|Necha2|102983       F---GLWKVDN--ATCADTVYEAIKAGYRLLDGACDYGNEKECGDGVARA 64
T.reesei|Trire_Chr|111218          F---GLWKVDN--AVCADTVYNAIKAGYRLFDGACDYGNEKECGEGVARA 63
B.cinerea_BC1T12462                F---GLWKVDN--ATCADTVYNAIKTGYRLFDGACDYGNEVEAGQGVARA 63
P.rubens_Pc16g09600                F---GLWKVNN--DTCADQVYHAIKAGYRLFDGACDYGNEVEAGQGVA-- 61
A.nidulans_AN0423                  F---GLWKVNN--DTCADQVYEAIKAGYRLFDGACDYGNEVEAGQGVA-- 61
A.niger|NRRL3|1952_XyrA            F---GLWKVNN--DTCADQIYHAIKEGYRLFDGACDYGNEVEAGQGIA-- 61
A.oryzae_AO090003000859            F---GLWKVNN--ETCADQVYEAIKAGYRLFDGACDYGNEVECGQGVA-- 61
B.cierea_BC1T00765                 F---GTFQNEGSPGKCHDAVLWALQSGYRHLDCAWYYKNEEEVGSGLREF 65
B.cinerea_BC1T04788                F---GTFASEGSVGETHKAVVAALNAGYRHLDCAWFYQNENEVGTAVKEF 70
A.niger|NRRL3|2193                 F---GTFANEGATGETYKAVTCALNTGYRHLDCAWYYLNEGEVGDAVKDF 66
A.oryzae_AO090003001045            F---GTFASEGSKGETYQAVTKALETGYRHLDCAWFYLNEDEVGDGIHDF 66
A.nidulans_AN5563                  F---GTFASEGASGETYRAVKKALEVGYRHLDCAWFYQNEDEVGDAVRDF 66
P.rubens_Pc22g04850                F---GTFASEGSKGETYAAVTAALNIGYRHLDCAWFYLNEDEVGEGIRDF 66
P.oryzae_MGG00097                  F---GTFAAEGQPGQTYAATKAALEAGYRHLDCAWFYQNEDEIGNAIADF 69
N.haematococca|Necha2|64813        F---GTFANEGAKGETYKAVTCALKAGYRHLDCAWFYLNEAEVGQAVRDF 67
P.nodorum_SNOG08193.3              F---GTFANEGSKGETYKAVTHALETGYRHLDCAWFYQNEEEVGDAVRDF 69
N.crassa_NCU04923                  F---GTFANEGAKGETYAAVKKALEVGYRHLDCAWFYQNEDEVGQALAEF 70
T.reesei|Trire_Chr|116623          F---GTFANEGAKGETYAAVTKALDVGYRHLDCAWFYHNEDEVGDAVRDF 65
Clustal Consensus                        :               *:  **  .* *  * *.   .. :

                                            160       170       180       190       200
                                   ....|....|....|....|....|....|....|....|....|....|
                                                      *
A.nidulans_AN1274                  --------SG-VPREEIWITTKLDNTW-HH---RVTDGINSSLKDLGVDY 103
A.oryzae_AO090038000442            --------SG-VPREEIWITTKLDNTW-HH---RVSEGIESSLKDLGVSY 104
A.niger|NRRL3|10868_XyrB           --------SG-VPREEIWVTTKLDNPW-HH---RVAEGIDSSLKDLGLDY 104
P.rubens_Pc16g05650                --------SG-VPREEIWLTTKLDNPW-HK---RVTEGIDSSLKDLQTDY 103
B.cinerea_BC1T03301                --------SG-VPREEIWLTTKLDNPW-HK---RVQEGIDSSLKSLGTDY 104
V.alfalfa|Veral1|1447              --------SG-VPRSEIWLTTKLDNPW-HK---RVQEGIASSLESLQTDY 104
P.nodorum_SNOG_14959.3             --------SG-IPREQIWITTKLDNPW-HK---RVEEGINSSLESLGVDY 104
N.haematococca|Necha2|73244        --------SG-VPREQIWLTTKLDNDC-HH---RVAEAIDTSLANLGTDY 104
N.crassa_NCU04510                  --------SG-VPREKIWLTTKLDNDW-HK---HVAEAIDTSLKNLDTPY 128
N.haematococca|Necha2|69859        --------SG-VPREEIWVTSKLRNNW-HK---HVREGLETSLKNLGLDY 100
N.haematococca|Necha2|79737        --------SG-TPREDIWVTTKLDNDW-HH---RASEGLASSLKDLGLDY 104
S.stipitis|Picst3|63050            --------SG-LKREEVFITTKLWNGD-HK---NAAKALDESLKKLGVDY 105
C.boidini|Canbo1|3509              --------SG-IPREELFVTTKLWCTY-HT---RVQQGLDESLERLGLEY 104
S.cerevisiae|Sacce1|5773_GCY1      --------SG-VPREEIFVTTKLWCTQ-HH---EPEVALDQSLKRLGLDY 105
S.cerevisiae|Sacce1|1403_YPR1      --------SG-VPREEIFITTKLWGTE-QR---DPEAALNKSLKRLGLDY 105
C.boidini|Canbo1|1386              --------SG-VPREELFITTKLWNTD-HR---KPLESLDASLKRLGLDY 103
S.stipitis|Picst3|88249            --------SG-IPREELYITTKLWCTK-HD---NPEAALDESLSNLGLDY 103
S.stipitis|Picst3|31015            --------SG-IPRKEIFVTTKLAPID-AL---DPAGALDQSLKNLGLDY 104
A.oryzae_AO090010000381            --------SG-VPREKIFITSKLWNTH-QP---NVAEGLQKTLNALGTDY 100
P.rubens_Pc12g04240                --------SG-VPRENIFITSKLWNTH-QP---NVGEGLQKTLDALGTDY 100
P.oryzae_MGG_02921                 I------SSGIVKREDLFVVSKCWATY-TT---RCELGLDQSLKLLGLDY 103
N.crassa_NCU01906                  F------DQGIVKREDVFVVTKLWATY-TS---RAEEGLEKSLRNLGLEY 103
N.haematococca|Necha2|67734        F------DAG-VKREDIFIVTKVWATY-NT---RCELALDKSLKALGLDY 102
T.reesei|Trire_Chr|113861          F------AAG-VKREDIFVVTKIWATY-NT---RVVLGLDKSLRSLGLDY 102
V.alfalfa|Veral1|4674              F------AAG-VKREDIFVTSKLWGTY-QTSDARVEEALDKSLKSLGLEY 105
B.cinerea_BC1T02386                F------ASG-VKREDIFITTKLWCTY-HT---RVEEALDKSLKRLGLDY 102
```

```
                                                          *
P.nodorum_SNOG07475.3            I------SRGIVKREELFITSKLWCTF-HT---RAEEGLQKSLDMLKTPY 125
A.nidulans_AN5986                L------SEGIVKRSEIFVTTKLWCTY-HT---RIQQALDLSLSKLGLDY 105
A.oryzae_AO090011000614          L------ASGKVKREDLFVTTKLWCTY-HS---RVEEALEKSLKNLGLDY 105
P.rubens_Pc22g20340              I------DAGIVKREDLFVTTKLWCSY-HA---RVEEGLQQSLTDLGLDY 105
A.niger|NRRL3|6930               I------ESGVVKREDLFVTTKLWSTW-HY---RVEQALDQSLKNLGLDY 109
A.niger|NRRL3|10050_LarA         Y----KDVPG-LKREDIFITSKLWNSQ-HDPA-VVEKALDECLAELELDY 107
P.rubens_Pc20g15580              Y----KDVPG-LKREDLFITSKLWNSQ-HRPE-VVEASLDACLAELELDY 107
A.nidulans_AN7193                Y----KDIPG-LKREDIFITSKLWNTQ-HDPA-VVEKALDDCLAELELDY 107
A.oryzae_AO090023000264          F----EEFN--IKREDVFITSKLWNSQ-HHPD-VVEKALDDCLAECGLDY 106
N.haematococca|Necha2|36240      F----KEIPG-LRREDVFITSKLWNSQ-HDPE-IVEAALDDCLEELGLEY 107
P.nodorum_SNOG_02188.3           FG---GAVPG-LKREDVFITSKLWNSQ-HRPQ-DVPAALDDCLAELGLDY 108
N.haematococca|Necha2|100823     L----AEVPG-LKREDIFITSKLWNNK-HRPE-EVAGALDDTLEELGLDY 107
T.reesei|Trire_Chr|110647        L----AEVPG-LKREDIFITSKLWNNS-HKPE-DVEPALDDTLAELGLDY 107
A.niger|NRRL3|7282               --------SG-VPREEIFLTSKLWNTH-HHPE-NVEEAVDKSLADLQTDY 102
S.cerevisiae|Saccel|3067         I------SEGLVSRKDIFVVSKLWNNF-HHPD-HVKLALKKTLSDMGLDY 104
C.boidini|Canbo1|6002            I------KDGLVKREELFIVSKLWNNF-HHPD-SVKLAIKKVLSDLNLEY 105
S.stipitis|Picst3|89614          I------DEGIVKREDLFLTSKLWNNY-HHPD-NVEKALNRTLSDLQVDY 103
N.haematococca|Necha2|38020      I------SEGLVKREDIFVTTKLWNNY-HKRE-HALAMAKLQNEAWGLGY 105
V.alfalfa|Veral1|9347            I------KEGLVKREDIFITTKLWNNY-HRKE-HAIDMARKQNEAWGLGY 105
P.oryzae_MGG01404                I------EEGIVKREDLFITSKLWNNY-HKHE-HAIEMAKHEVDTWGIGY 105
V.alfalfa_|Veral1|801            I------AHGLVSRDDLFITSKLWNNH-HAPA-HATRMVNEELRAWGLDQ 105
P.nodorum_SNOG_12824.3           I------KEGLVKREDLFIVSKLWQTF-HDYE-QVEPITKKQLKDWGIDY 162
P.oryzae_MGG03648                I------DEGLVKREELFIVSKLWNTF-HDGE-RVEPIVKKQLADWGIEY 105
N.crassa_NCU08384                I------KEGIVKREELFIVSKLWNTF-HDGD-RVEPIVRKQLADWGLEY 104
N.haematococca|Necha2|102983     I------KEGIVKREDLFIVSKLWQTY-HEEQ-HVEPITRRQLADWQVDY 106
T.reesei|Trire_Chr|111218        I------KDGLVKREDLFIVSKLWQTF-HDED-KVEPITRRQLADWQIDY 105
B.cinerea_BC1T12462              IK------EGLVKREDLFIVSKLWNSF-HDGD-RVGPITRKQLADWGIDY 105
P.rubens_Pc16g09600              -RAI---KEGIVKREELFIVSKLWNSF-HEAD-KVEPIARKQLADWGVDY 105
A.nidulans_AN0423                -RAI---KEGIVKRSDLFIVSKLWNSF-HDGE-RVEPIARKQLSDWGIDY 105
A.niger|NRRL3|1952_XyrA          -RAI---KDGLVKREELFIVSKLWNSF-HDGD-RVEPICRKQLADWGIDY 105
A.oryzae_AO090003000859          -RAI---KEGIVKREELFIVSKLWNSF-HEGD-RVEPICRKQLADWGVDY 105
B.cierea_BC1T00765               L----SSNPS-VKRSDIFITTKVWPHLCGSPE-DVEWSLNYSLEKLGVDY 109
B.cinerea_BC1T04788              L----AANPS-VKRSDIFIVTKVWNHL-HDPE-DVEWSLKNSLEKLQTPY 113
A.niger|NRRL3|2193               L----AANPS-VKREDIFICTKVWNHL-HRPE-DVQWSIESSLKKLKVDY 109
A.oryzae_AO090003001045          L----KKNPS-VKREDIFVCTKVWNHL-HRPE-DVQWSVDNSLKRLRLDY 109
A.nidulans_AN5563                L----KENPS-VKREDIFICTKVWNHL-HRPE-DVRWSIEDSLKKLKTDY 109
P.rubens_Pc22g04850              L----KANPS-VKRSDIFVTTKVWNHL-HRYD-DVLWSINDSLERMKLDY 109
P.oryzae_MGG00097                L----KENPS-VKREDLFICTKVWNHM-HAPE-DVKWSLDNSLKALRLDY 112
N.haematococca|Necha2|64813      L----SENPS-VKREDLFITTKVWNHL-HEPE-DVKWSMQNSLSNFGLDY 110
P.nodorum_SNOG08193.3            L----KANPS-VTRKDLFICTKVWQHL-HEPD-EVEWSFNSSLQKLQMDY 112
N.crassa_NCU04923                L----ENHKD-VKREDIFICTKVWNHL-HEPE-DVKWSLQNSLDKLKVDY 113
T.reesei|Trire_Chr|116623        L----ARRPD-VKREDLFICTKVWNHL-HEPE-DVKWSAKNSCENLKVDY 108
Clustal Consensus                            *..:::  :*

                                         210        220        230        240        250
                                 ....|.*..|....|....|....|....|....|....|....|....|
A.nidulans_AN1274                VDLYLMHWPSSTD----PND--------------------LKK----H-L 124
A.oryzae_AO090038000442          VDLYLVHWPSSTD----PND--------------------KSK----H-L 125
A.niger|NRRL3|10868_XyrB         VDLYLVHWPSSTD----PND--------------------LKK----H-L 125
P.rubens_Pc16g05650              VDLYLMHWPSSTD----PND--------------------LSK----H-L 124
B.cinerea_BC1T03301              VDLYLMHWPSSTD----PDD--------------------LKK----H-Y 125
V.alfalfa|Veral1|1447            VDLYLMHWPSSTD----PED--------------------LKK----H-H 125
P.nodorum_SNOG_14959.3           VDLYLMHWPSSTD----PED--------------------LKK----H-L 125
N.haematococca|Necha2|73244      VDLYLMHWPVALD----PGN--------------------HDQ----V-L 125
N.crassa_NCU04510                VDLYLMHWPASLV----KGN--------------------TKE----V-Y 149
N.haematococca|Necha2|69859      VDLYLIHWPCSRD----ADD--------------------PAN----L-Y 121
N.haematococca|Necha2|79737      VDLFLVHFPCSTD----PTD--------------------SSK----H-L 125
S.stipitis|Picst3|63050          VDLYLIHWPVSTD------------PAT-------------GKD-----Y 125
C.boidini|Canbo1|3509            VDLYLIHWPFAMN----PNGADNWLPFK------EDG---VTRD-----V 136
S.cerevisiae|Saccel|5773_GCY1    VDLYLMHWPARLDPAYIKNEDILSVPTK------KDG----SRA---VDI 142
S.cerevisiae|Saccel|1403_YPR1    VDLYLMHWPVPLKTDRVTDGNVLCIPTL------EDG----TVD---IDT 142
```

```
                                            *
C.boidini|Canbo1|1386              VDLYLMHWPVPFHPAKTEEEDQFFVVNK------STG----EYD---NDL 140
S.stipitis|Picst3|88249            VDLYLIHWPVFLN----PNGNDPKFPTL------PNG----KRD---I-V 135
S.stipitis|Picst3|31015            VDLYLMHWPVCLN---KANKLHPGIPTL------PNG----KRD---I-V 137
A.oryzae_AO090010000381            LDLYLIHWPVRLV----PNESSELLPVN------PDG----TRS---V-D 132
P.rubens_Pc12g04240                LDLYLIHWPVRLV----PNESSALLPVN------PDG----TRS---V-D 132
P.oryzae_MGG_02921                 VDLYLVHWPILMN----PEGNDEKFPKH------ADG----SRD---I-I 135
N.crassa_NCU01906                  VDLFLVHWPLLMN----PEGNDDRFPKL------PNG----ERD---I-L 135
N.haematococca|Necha2|67734        VDLFLVHWPLLLN----PEGNHDKFPTK------EDG----SRD---V-I 134
T.reesei|Trire_Chr|113861          VDLLLVHWPVLLN----PEGNHDKFPTL------PDG----KRD---V-I 134
V.alfalfa|Veral1|4674              LDLYLIHWPVAMN----PEGNHDRFPTL------PDG----SRD---L-I 137
B.cinerea_BC1T02386                VDLYLMHWPVPMN----PDGNHELFPKH------PDG----SRD---L-Q 134
P.nodorum_SNOG07475.3              VDLFLVHWPVPMN----PKGNHPLFPKL------EDG----SRD---I-D 157
A.nidulans_AN5986                  VDLYLVHWPLAMN----PNGNHDLFPKL------PDG----SRD---L-V 137
A.oryzae_AO090011000614            IDLYLMHWPLAMN----PNGNHPLFPKH------EDG----SRD---I-D 137
P.rubens_Pc22g20340                VDLYLMHWPLAMN----PKGNHNLFPKL------ADG----SRD---I-V 137
A.niger|NRRL3|6930                 VDLYLVHWPVAMN----PNGNHPNIPTL------PDG----SRD---L-H 141
A.niger|NRRL3|10050_LarA           LDLYLVHWPVSFTT------GSELFPLVKDSSVEGG----DVV------I 141
P.rubens_Pc20g15580                LDLYLVHWPVAFQK------GDSYFPLVANSPVEGG----DVI------I 141
A.nidulans_AN7193                  LDLYLVHWPVAFKT------GDNYFPLDESSDHPDG----DVL------I 141
A.oryzae_AO090023000264            LDLYLVHWPVAFKT------GNEYFPLVEGSTVPGG----DCI------I 140
N.haematococca|Necha2|36240        LDLYLVHFPISFKKSS-VHVGQDLFPLTGGNQ-PEG----DVV------I 145
P.nodorum_SNOG_02188.3             LDLYLVHFPVAFDEPS-KP-HSQLFPLV-----ESG----DVK------M 141
N.haematococca|Necha2|100823       LDLYLIHWPVAFKN------GTELFPRKEG---DDS----QVA------I 138
T.reesei|Trire_Chr|110647          LDLYLIHWPVAFAP------GADLFPKSE----DGS----EVQ------L 137
A.niger|NRRL3|7282                 LDLYLIHWPVAFR---YSTTTIQ--PVNE-----QTG----LID-----V 133
S.cerevisiae|Sacce1|3067           LDLYYIHFPIAFK---YVPFEEKYPPGFYT---GAD----DEKK---GHI 141
C.boidini|Canbo1|6002              IDLFYMHFPIAQT---FVPIEKQYPPNFYC---GDG----DK-------W 138
S.stipitis|Picst3|89614            VDLFLIHFPVTFK---FVPLEEKYPPGFYC---GKG----DN-------F 136
N.haematococca|Necha2|38020        IDLYLIHFPVSLE---YIDPATRRFPAWWM---D-E----QGT------V 138
V.alfalfa|Veral1|9347              IDLFLIHFPCALK---YIEPSKLPYPAWWT---DAE----RSS------V 139
P.oryzae_MGG01404                  LDLFLIHFPISLE---YISHSKMPYPCFWP---DRE----KSR------S 139
V.alfalfa_|Veral1|801              LDLYLIHFPIATQ---WVDPSVSRFPSWHA---DAA----KTR------L 139
P.nodorum_SNOG_12824.3             FDLYLIHFPVALK---YVSPETRYPPGWFS---DEA----NSK------V 196
P.oryzae_MGG03648                  FDLYLIHFPVALE---YVDPSVRYPPGWHY---DDA----GTE------I 139
N.crassa_NCU08384                  FDLYLIHFPVALE---YVDPSVRYPPGWHF----DG----KSE------I 137
N.haematococca|Necha2|102983       FDLFLIHFPVALE---YIDPSVRYPPGWHI---DDA----QTE------I 140
T.reesei|Trire_Chr|111218          FDLFLVHFPAALE---YVDPSVRYPPGWFY----DG----KSE------V 138
B.cinerea_BC1T12462                FDLFIVHFPVALR---YVDPAVRYPPGWQYSDKEGD-------------- 138
P.rubens_Pc16g09600                FDLYIVHFPIALK---YLDPSVRYPPSW-----TTA----EGK------I 137
A.nidulans_AN0423                  FDLYIVHFPVSLK---YVDPEVRYPPGW-----ENA----EGK------V 137
A.niger|NRRL3|1952_XyrA            FDLYIVHFPISLK---YVDPAVRYPPGW-----KSE----KDE------L 137
A.oryzae_AO090003000859            FDLYIVHFPVALK---YVDPAVRYPPGW-----NSE----SGK------I 137
B.cierea_BC1T00765                 VDSFLMHWPFAAE-----RTDDYEV-KIG-----DDGKYIINKE-----V 143
B.cinerea_BC1T04788                IDAFLVHWPIAAE-----KNEDRSV-KIG-----ADGKYVIKKD-----L 147
A.niger|NRRL3|2193                 IDLFLVHWPIAAE----KETQEKP--KIG-----PDGKYVILKD-----L 143
A.oryzae_AO090003001045            VDLFLVHWPIASE----KEDQEKP--KIG-----PDGKYVILKE-----L 143
A.nidulans_AN5563                  VDLFLIHWPIAAE----KESQDKP--KIG-----PDGKYVILKD-----L 143
P.rubens_Pc22g04850                VDLFLVHWPIAAE----KDGQEKP--KIG-----PDGKYVILKD-----L 143
P.oryzae_MGG00097                  VDLFLVHWPIAAE-----RTEDRQV-KLG-----PDGKYVINHE-----L 146
N.haematococca|Necha2|64813        VDLFLVHWPIAAE-----KDADNKP-KIG-----PDGKYVIKKD-----L 144
P.nodorum_SNOG08193.3              IDLFLVHWPIAAE-----SNSDHMP-KLG-----ADGKYIIKKD-----L 146
N.crassa_NCU04923                  VDLFLIHWPIAAE-----KDEATNMPKIG-----PDGKYIIKKE-----L 148
T.reesei|Trire_Chr|116623          IDLFLVHWPIAAE-----KNSDRSV-KLG-----PDGKYVINQA-----L 142
Clustal Consensus                  .*    :*:*                                      24

                                            260       270       280       290       300
                                   ....|....|....|....|....|....|....|....|....|....|
A.nidulans_AN1274                  ---PDWDFIKTWQEMQKLPA-----TG--KVRNIGVSNFGIRNLEKLLND 164
A.oryzae_AO090038000442            ---PDWDFIKTWQEMQKLPA-----TG--KVRNIGVSNFGIKNLEKLLND 165
A.niger|NRRL3|10868_XyrB           ---PDWDFIKTWQEMQKLPA-----TG--KVRNIGVSNFGIKNLEKLLND 165
P.rubens_Pc16g05650                ---PDWDFIKTWQELQKLPA-----TG--KVRNIGVSNFGIKNLEILLND 164
```

```
B.cinerea_BC1T03301               ---PDWDFIKTWHEMQKLPA-----TG--KVKNIGVSNFGIKNLEKLLSD 165
V.alfalfa|Veral1|1447             ---PDWDFVDTWREMQKLVG-----TG--KVRNIGVSNFGIKNLERLLND 165
P.nodorum_SNOG_14959.3            ---PDWDFLDTWAELQKLPE-----SG--RVKNIGVSNFAVKNLEKLFAD 165
N.haematococca|Necha2|73244       ---KDWDFVDTWRDMQKLVG-----TG--KVRNIGVSNFGIRNLDKLLSA 165
N.crassa_NCU04510                 ---NDWDFVDTWREMQKLVD-----TG--KVKNIGVSNFGVKNLEKLLSA 189
N.haematococca|Necha2|69859       ---RDWDFVDTWAEMQRLLD-----GG--KVRNIGVSNFGIRHLEELLNS 161
N.haematococca|Necha2|79737       ---TDWDYVKTWQEMQKLLD-----TG--KVKNIGVSNFQIRHLEKLLSD 165
S.stipitis|Picst3|63050           ---EDWDYVDTWKQLQKLYK----ETK--KVRAIGVSNFTKSKIERLLAD 166
C.boidini|Canbo1|3509             ---IEWDYVKTYEAMQELLP-----SG--KVRAIGISNFTEEKIERLLNS 176
S.cerevisiae|Sacce1|5773_GCY1     ---TNWNFIKTWELMQELPK-----TG--KTKAVGVSNFSINNLKDLLAS 182
S.cerevisiae|Sacce1|1403_YPR1     ---KEWNFIKTWELMQELPK-----TG--KTKAVGVSNFSINNIKELLES 182
C.boidini|Canbo1|1386             ----EWDFIKTYELMQELVP-----TG--KTRSVGVSNFSKTNLEKLLNS 179
S.stipitis|Picst3|88249           ---TDWNFVKTYELLQPLVA-----LG--KTKSIGVSNFSVTNLEKLLNA 175
S.stipitis|Picst3|31015           ---FDRDFTQTYADMQHLVE-----SG--KAKSIGVSNFSIKNLKKLFSS 177
A.oryzae_AO090010000381           ---RSWDQSETWRQMEEVYK-----SG--KVKAIGVANWSIPYLEELK-- 170
P.rubens_Pc12g04240               ---RSWDQSETWRQMEDVYK-----AG--KVKAIGVANWSIPYLEELK-- 170
P.oryzae_MGG_02921                ---HTHNHVDTWKLMEKLPA-----TG--KTKAVGVSNYSKAWLEQLL-- 173
N.crassa_NCU01906                 ---RDYSHVQIWKNMEKLVG-----SG--RTKAIGVSNYSKRYLEELL-- 173
N.haematococca|Necha2|67734       ---RSYNHVDGWKQMEKLPA-----TG--KTKGVGVCNYSKRYLEELL-- 172
T.reesei|Trire_Chr|113861         ---WDYNHVDGWKQMEAVLA-----TG--KTKSIGVSNYSKKYLEQLL-- 172
V.alfalfa|Veral1|4674             ---RDHKHTDTWKSMEKLLD-----TG--KVKAIGVCNYSKRYLEELL-- 175
B.cinerea_BC1T02386               ---TEWSHTQTWQELEKVSK-----TG--KTKAIGVSNYSVKNLEELF-- 172
P.nodorum_SNOG07475.3             ---HSITHIQTWQNMEKLIQ-----SNPDKVKAIGVANYSVKYMEKLL-- 197
A.nidulans_AN5986                 ---REHSHVTTWKGMEELIT-----NNPDKVKAIGVSNYSKRYLEQLL-- 177
A.oryzae_AO090011000614           ---HSHSHVQTWKNMEKLLA-----TG--KVKAIGVSNYSVRYLEQLL-- 175
P.rubens_Pc22g20340               ---HSHSHVTTWKSMEKLVG-----TG--KVKAIGVSNYSVKFLEELL-- 175
A.niger|NRRL3|6930                ---LNHSHINTWKDMEKLVG-----SG--KTKAIGVCNYSRPYLEELL-- 179
A.niger|NRRL3|10050_LarA          -N-DDISIVDTWKAMTQLPK--------SKARTVGVSNHMIPHLEAII-- 179
P.rubens_Pc20g15580               -D-DGVSIVDTWKAMTQLPK--------NKARSVGVSNHKIEHLEALI-- 179
A.nidulans_AN7193                 -D-DSISIVDTWKAMTKLPK--------EKARAIGVSNHTVEHLEAII-- 179
A.oryzae_AO090023000264           -D-DSISIVDTWKAMTKLPK--------SKARAIGVSNHMVEHLEAII-- 178
N.haematococca|Necha2|36240       -D-DTISIVDTWRAMTKLPK--------SKARAIGVSNHTVEHLKAII-- 183
P.nodorum_SNOG_02188.3            -L-DDISIVDTWKAMTKLPK--------EKARAVGVSNHTKEHLQALI-- 179
N.haematococca|Necha2|100823      -D-RGVSLSQTWKAVTELPK--------SKVRSIGVSNFSIEHLETVI-- 176
T.reesei|Trire_Chr|110647         -N-QNVSIVQTWKAMTELPK--------SKVRSVGVSNFTIEHLDAVI-- 175
A.niger|NRRL3|7282                ---VDVPIKDTWAAMEKLVE-----KG--KVRSIGVSNFTREKIEELL-- 171
S.cerevisiae|Sacce1|3067          TE-AHVPIIDTYRALEECVD-----EG--LIKSIGVSNFQGSLIQDLL-- 181
C.boidini|Canbo1|6002             SF-ENVPISTTWKAMEELVE-----QG--LVKSIGISNFVGALIQDLL-- 178
S.stipitis|Picst3|89614           DY-EDVPILETWKALEKLVK-----AG--KIRSIGVSNFPGALLLDLL-- 176
N.haematococca|Necha2|38020       KP-DNTPIRETWEALETVVD-----EG--IARSIGVSNFQAQSLYDLQ-- 178
V.alfalfa|Veral1|9347             ET-DKVPIRETWESLESLVD-----EG--VAKSIGVSNFQAQSLYNVF-- 179
P.oryzae_MGG01404                 TPLQYTPVAETWAALESLVKTDSNPDG--ILRSIGVANFRAQLLTDLW-- 185
V.alfalfa_|Veral1|801             HPRARVPLADTWRALEALVDAPGVRAG--PVRSIGVSNFDAQLLYDLL-- 185
P.nodorum_SNOG_12824.3            IH-SKARLEDTWRAFEDIKS-----KG--LTKSIGVSNYSGALLLDLF-- 236
P.oryzae_MGG03648                 RP-SKASIQETWTAMEKLVD-----AG--LSKAIGVSNFQAQLLYDML-- 179
N.crassa_NCU08384                 RP-SKATIQETWTAMESLVE-----KG--LSKSIGVSNFQAQLLYDLL-- 177
N.haematococca|Necha2|102983      RW-GKATNQETWGAMEKLVE-----KG--LAKSIGISNFQSQAIYDLL-- 180
T.reesei|Trire_Chr|111218         RWSKTTTLQQTWGAMERLVD-----KG--LARSIGVSNYQAQSVYDAL-- 179
B.cinerea_BC1T12462               VQQSKASIQETWQAMEKLVDEG-------LAKSIGVSNFQGALLIDLLR- 180
P.rubens_Pc16g09600               EF-ANAPIHETWGAMETLVD-----KK--LARSIGVSNFSAQLLMDLL-- 177
A.nidulans_AN0423                 EL-GKATIQETWTAMESLVD-----KG--LARSIGISNFSAQLLLDLL-- 177
A.niger|NRRL3|1952_XyrA           EF-GNATIQETWTAMESLVD-----KK--LARSIGISNFSAQLVMDLL-- 177
A.oryzae_AO090003000859           EF-SNATIQETWTAMESLVD-----KK--LARSIGVSNFSAQLLMDLL-- 177
B.cierea_BC1T00765                ----TANLEPIWRRFEALNK-----AG--KAKAIGVSNFTISNLQALL-- 180
B.cinerea_BC1T04788               ----TENPEPTWRAMEKLYK-----EG--LAKAIGVSNWTEKGIEQLL-- 184
A.niger|NRRL3|2193                ----TENPEPTWRAMEKIYK-----EK--KAKAIGVSNWTIEGLEKLL-- 180
A.oryzae_AO090003001045           ----TENPEPTWRAMEKIYR-----DG--KAKAIGVSNWTIPGLEKLF-- 180
A.nidulans_AN5563                 ----TEDPKPTWQAMEKLYE-----DK--LARSIGVSNWTIEGLEKLL-- 180
P.rubens_Pc22g04850               ----TENHEETWRAMEKLYA-----DG--KAKAIGVSNWTIPQLEAMA-- 180
P.oryzae_MGG00097                 ----TENPEPTWRAMEELYE-----AK--KARAIGVSNWTIDGLKKLF-- 183
N.haematococca|Necha2|64813       ----TENPEPTWRAMEELLA-----SG--KTRAIGVSNWTITGLKKLL-- 181
```

```
P.nodorum_SNOG08193.3            ----TENPEPTWRAMEALNK-----AG--KAKAIGVSNWTIEGLKQLM-- 183
N.crassa_NCU04923                ----TENPEPTWRAMEDLVD-----AG--KTRSIGVSNWTIPGLQKLL-- 185
T.reesei|Trire_Chr|116623        ----TENPEPTWRAMEELVE-----SG--LVKAIGVSNWTIPGLKKLL-- 179
Clustal Consensus                           :  .                : :*:.*      :

                                         310       320       330       340       350
                                 ....|....|....|....|....|....|....|....|....|....|
A.nidulans_AN1274                PSCKIVPAVNQIELHPNNPSPKLVAYNSSKGIHSTGYSCLGST------- 207
A.oryzae_AO090038000442          PSTKIVPAVNQIELHPNNPSPKLVAYNTSKGIHSTGYSCLGST------- 208
A.niger|NRRL3|10868_XyrB         PSCKIVPAVNQIELHPNNPSPKLVAYNTSKGIHSTGYSCLGST------- 208
P.rubens_Pc16g05650              PSCKIVPAVNQIELHPNNPSPKLVAYNTSKGIHSTGYSCLGST------- 207
B.cinerea_BC1T03301              PGTTIVPAVNQIELHPNNPSPKLVAYNTSKGIHSTGYSCLGST------- 208
V.alfalfa|Veral1|1447            PSCKIVPAVNQIELHPCNPSPKLLEYNASKGIHSTAYSCLGST------- 208
P.nodorum_SNOG_14959.3           SRFKTTPAVNQIELHPNNPSPKLLDYCKQKGIHATAYSCLGST------- 208
N.haematococca|Necha2|73244      ESCKITPAVNQVELHPFNPSTRLVKYNESKGIHTTAYSPLGST------- 208
N.crassa_NCU04510                ESTKIVPAVNQIELHPGNPSPHLVEYLRSKGIHASAYSPLGSS------- 232
N.haematococca|Necha2|69859      PSTKVVPAVNQIELHPCNPSPKLVAYNTSKGIHTEGYSCLGSA------- 204
N.haematococca|Necha2|79737      PSCKVVPAVNQLELHPYNPSPKLVEYCKSKGIHCTAYSCLGGNSTSPIN- 214
S.stipitis|Picst3|63050          PEVDVVPAVNQVEAHPLLTQPELYDYLKEKNIVIEAYSPLGST------- 209
C.boidini|Canbo1|3509            ESTKIKPACLQIELHPLLPQQKLIDYCKSKDIVVEAYSPLGST------- 219
S.cerevisiae|Sacce1|5773_GCY1    QGNKLTPAANQVEIHPLLPQDELINFCKSKGIVVEAYSPLGST------- 225
S.cerevisiae|Sacce1|1403_YPR1    PNNKVVPATNQIEIHPLLPQDELIAFCKEKGIVVEAYSPFGSA------- 225
C.boidini|Canbo1|1386            ANLKIKPACNQVELHPFLPQFDLLEFCKTNDIQLEAYAPLGSH------- 222
S.stipitis|Picst3|88249          PTTKIVPVVNQVELHPYLPQQQLLEYTKKHGIVLEAYSPLGST------- 218
S.stipitis|Picst3|31015          PDYKIVPTVNQVEIHPYLPQTELLEFCKKHDILLEAFSPLGSS------- 220
A.oryzae_AO090010000381          KKWTVVPAVNQVELHPFLPQHALKEWCDKHGILLEAYSPLGSE------- 213
P.rubens_Pc12g04240              KKWTVVPAVNQVELHPFLPQHALKDWCDKHGILLEAYSPLGSE------- 213
P.oryzae_MGG_02921               PHATTVPAVNQVENHPQLPQQELVDFCKEKGIHIMAYSPLGST------- 216
N.crassa_NCU01906                PHAKIVPAVNQIENHPQLPQQEIVDFCKEKGIHIMAYSPFGST------- 216
N.haematococca|Necha2|67734      PHATIIPAVNQIENHPSLPQQEIVDFCKEKGIHIEAYSPLGST------- 215
T.reesei|Trire_Chr|113861        PHATVIPAVNQIENHPSLPQQEIVDFCKEKGIHIMAYSPLGST------- 215
V.alfalfa|Veral1|4674            PVAKVIPAVNQIENHPSLPQDEIVSLCKEKGIQIMSYSPFGST------- 218
B.cinerea_BC1T02386              SKATITPAVNQIENHPSLPQQEIYDLCKSKGIHITAYSPLGST------- 215
P.nodorum_SNOG07475.3            AKATIVPAANQIENHPLLPQQEVVDFCKSKGIHITAYSPLGST------- 240
A.nidulans_AN5986                PQAKIVPAVNQIENHPALPQQEIVDLCKEKGILITAYSPLGST------- 220
A.oryzae_AO090011000614          PEATVVPAVNQIENHPSLPQQEIVDFCKKKGIHITAYSPLGST------- 218
P.rubens_Pc22g20340              PQATIIPAANQIENHPLLPQQEIVDFCNKAGIHITAYSPLGST------- 218
A.niger|NRRL3|6930               AQATVVPAVNQIENHPCLPQQEAVDFCKEKGIHITAYSPLGST------- 222
A.niger|NRRL3|10050_LarA         NATGVVPAVNQIERHPVLQSNELIEYCQKKGIHVTAYSAFGNNGF----- 224
P.rubens_Pc20g15580              KGTGVVPAANQIERHPVLQSNDLIEYCQQKGIHVTAYSAFGNNML----- 224
A.nidulans_AN7193                NATGVVPAANQIERHPVLQSNDLIEYAAKKNIHITAYSAFGNNMF----- 224
A.oryzae_AO090023000264          NATGVVPAANQIERHPVLQSPKLIQYCKEKGIHVTAYSAFGNNMI----- 223
N.haematococca|Necha2|36240      DGTSVVPAVNQIERHPLLRQDDLIAFCKEKNIHITAYSAFGNNML----- 228
P.nodorum_SNOG_02188.3           DGSGITPAANQIERHPRLLQPELIQFCKEKNIHITEYSAFGNNML----- 224
N.haematococca|Necha2|100823     KDTGVVPAVNQVERHPRLPNPELVAYLKEKGIVLTAYSAFGNNSW----- 221
T.reesei|Trire_Chr|110647        EATGVVPAVNQIERHPRLPNQPLIDYCAKKGIIITAYSAFGNNTK----- 220
A.niger|NRRL3|7282               KTAKITPAVNQIEAHPFLQQRDLLEWSTQKGIVVAGYSPLGNNIY----- 216
S.cerevisiae|Sacce1|3067         RGCRIKPVALQIEHHPYLTQEHLVEFCKLHDIQVVAYSSFGPQSFIEMD- 230
C.boidini|Canbo1|6002            RGCKIRPAVLQIEHHPYLVQPRLIEYAKAEGINVTAYSSFGPQSFVELD- 227
S.stipitis|Picst3|89614          RGATIKPSVLQVEHHPYLQQPRLIEFAQSRGIAVTAYSSFGPQSFVELN- 225
N.haematococca|Necha2|38020      RYARHPVSSLQIEHHPYLVQPDLIAMAQENKIAVTAYSSFGPQSFKELPG 228
V.alfalfa|Veral1|9347            TYNRHPLSSLQIEHHPYLVQPELVQLAQENKIAVTAYSSFGPQSFMELPP 229
P.oryzae_MGG01404                GSAKIKPAVNQIEHHPYLVQPQLLAFLKDHGIAITAYSSFGPQSFVELD- 234
V.alfalfa_|Veral1|801            TYARVPPAVLQVEHHPYLAQPQLVAVARESGIAVTAYSTFGPQSFLELG- 234
P.nodorum_SNOG_12824.3           TYAKVKPATLQIEHHPYYVQPYLIKLAEEHDIKVTAYSSFGPQSFIECD- 285
P.oryzae_MGG03648                RYARIRPATLQIEHHPYLVQQRLIEACKTEGIVVTAYSSFGPASFKEFN- 228
N.crassa_NCU08384                RYAKVRPATLQIEHHPYLVQQNLLNLAKAEGIAVTAYSSFGPASFREFN- 226
N.haematococca|Necha2|102983     KYAKIRPATLQVELHPYYQQTELVRLAKAEGIALTAYSSFGPAGFIELD- 229
T.reesei|Trire_Chr|111218        IYARIKPATLQIEHHPYLQQPDLVSLAQTEGIVVTAYSSFGPTGFMELD- 228
B.cinerea_BC1T12462              -YARIRPATLQIEHHPYLVQNTLLKLAESEGIKVTAYSSFGPQSFIELG- 228
P.rubens_Pc16g09600              RYARVRPATLQIEHHPYLTQTRLVDYAQKEGITVTAYSSFGPLSFLELD- 226
```

```
A.nidulans_AN0423               RYARIRPATLQIEHHPYLTQERLVTFAQREGIAVTAYSSFGPLSFLELS- 226
A.niger|NRRL3|1952_XyrA         RYARIRPATLQIEHHPYLTQTRLVEYAQKEGLTVTAYSSFGPLSFLELS- 226
A.oryzae_AO090003000859         RYARVRPATLQIEHHPYLTQPRLVEYAQKEGIAVTAYSSFGPLSFLELE- 226
B.cierea_BC1T00765              KYADVPPAINQVEIHPVWPNTKLINYCFSKNILPVAYSPLGSQSQVPTT- 229
B.cinerea_BC1T04788             SFAEVKPTINQIEIHPFLPQQKLIDYCLSKDIVPVAYSPLGSQDQVPGT- 233
A.niger|NRRL3|2193              KYAEVKPHVNQIEIHPFLPNQELVDYCFKHDILPEAYSPLGSQNQVPTT- 229
A.oryzae_AO090003001045         KFAEIKPHVNQIEIHPFLPNNELVQFCFKNDILPEAYSPLGSQNQVPTT- 229
A.nidulans_AN5563               KYAKVKPHVNQIEIHPFLPNEELIQYCWKNDILPEAYSPLGSQNQVPTT- 229
P.rubens_Pc22g04850             KFAKVQPMVNQIEIHPFLANDELVQYCFSHNILPQAYSPLGSQNQVPTT- 229
P.oryzae_MGG00097               AVAKVKPAVNQIEIHPYLPNEELVRFCLDNDVLPSAYSPLGSQDQVPTT- 232
N.haematococca|Necha2|64813     GFAKVKPAVNQIEIHPFLPNTELVKFCQDNGILPQAYSPLGSQDQVPTT- 230
P.nodorum_SNOG08193.3           KFAEIKPAVNQIEIHPFLPNTELVKFCLDNDIMPAAYSPLGSQNQVPST- 232
N.crassa_NCU04923               KFARIKPTVNQIEIHPFLPNTELVEFCFKNQIIPTAYSPLGSQNQVPST- 234
T.reesei|Trire_Chr|116623       QIAKIKPAVNQIEIHPFLPNEELVAFCFENGILPEAYSPLGSQNQVPST- 228
Clustal Consensus                         *:* **   .           :    ::  :*

                                        360       370       380       390       400
                                ....|....|....|....|....|....|....|....|....|....|
A.nidulans_AN1274               -------NSP-LYKDPTLLKLAE------KK-GKTPQQVLLVWGIQKGWS 242
A.oryzae_AO090038000442         -------NSP-LYKDPTLLQLAE------KK-GKTPQQCLLQWGIQKGWS 243
A.niger|NRRL3|10868_XyrB        -------NSP-LYKDETLLKLAE------KK-GKTPQQVLLLWGVQKGWS 243
P.rubens_Pc16g05650             -------NSP-LYKDPTLLQIAE------KK-GKTPQQVLLVWGIQKGWS 242
B.cinerea_BC1T03301             -------NSP-LYKDQTLLSLAE------AK-GKTPQQVLLAWGIQKGWS 243
V.alfalfa|Veral1|1447           -------DSP-LYKNETLLNIAK------AK-GKTPQQVLLLWGLSRGSS 243
P.nodorum_SNOG_14959.3          -------DSP-LYKNEKLKKLAE------NK-GKSVQQVLLMWGLQRGSS 243
N.haematococca|Necha2|73244     -------NSP-IYSNETVISIAK------TH-GRTPQQILLMWGLQRGTS 243
N.crassa_NCU04510               -------DSP-LYKLNSLTKLAE------SK-GKTVQQVLLRWGVQKGWS 267
N.haematococca|Necha2|69859     -------HLG-LSRNKILVDIAN------AK-GKTPQQVLLQWGLQKGWS 239
N.haematococca|Necha2|79737     ------GHTD-LVKNGTVAKIAE------AK-EKTPAQILLKWGLQRGTS 250
S.stipitis|Picst3|63050         -------DAP-LFKNETIAAIAK------KN-GVEPGHVLISWAVQRNTV 244
C.boidini|Canbo1|3509           -------GAP-LLKNEKLIKIAE------KY-GVSPATICISWAIWRGTV 254
S.cerevisiae|Sacce1|5773_GCY1   -------DAP-LLKEPVILEIAK------KN-NVQPGHVVISWHVQRGYV 260
S.cerevisiae|Sacce1|1403_YPR1   -------NAP-LLKEQAIIDMAK------KH-GVEPAQLIISWSIQRGYV 260
C.boidini|Canbo1|1386           -------DSN-LMNDDIIKTLSE------KL-DVSPATLLVSWAVWRGTV 257
S.stipitis|Picst3|88249         -------NSP-LFKDETVVKIAE------KN-GVSPATILISWALWRGTV 253
S.stipitis|Picst3|31015         -------NSP-LLKDETIVKIAE------KN-QVSVATILISWAIWRGTV 255
A.oryzae_AO090010000381         -------GAP-LMSDPVIQEIAK------KN-GVSAATILISYHVNRGVV 248
P.rubens_Pc12g04240             -------GAP-LMSDPAIQEIAN------KN-DVSPATILISYHVNRGVV 248
P.oryzae_MGG_02921              -------GGP-LLTAEPVVKIAE------KH-SISPAAVLLGYQIARGIT 251
N.crassa_NCU01906               -------GSP-VTSAEPVIKIAE------KH-GVKPTTVLLSYHLYRGST 251
N.haematococca|Necha2|67734     -------GGP-VMTAEPVVKIAE------KK-GVSASTVLLSYH---GNT 247
T.reesei|Trire_Chr|113861       -------GSP-LMSADPVVKIAE------KK-GISPTTVLLSYHVNRGST 250
V.alfalfa|Veral1|4674           -------GGP-LMSAPVIVKIAE------KH-GVSPGTVLLSYNTSRGST 253
B.cinerea_BC1T02386             -------GSP-LFTAAPIVEVAK------KR-DVPPASILLSYHVARGSS 250
P.nodorum_SNOG07475.3           -------GSP-LFKDEGILEVAK------KH-DVGPATVLLSYHLARGAS 275
A.nidulans_AN5986               -------GSP-LFKAEAIVAVAE------RR-GVTPASVLLSWHLARGSS 255
A.oryzae_AO090011000614         -------GSP-LFTAEPIVEVAK------KK-GVTPATVLLSWHIARGSS 253
P.rubens_Pc22g20340             -------GSP-LFTAEPIVAVAN------KR-GVTPATVLLSWHIARGSS 253
A.niger|NRRL3|6930              -------GSP-LLTAEPIVEVAK------KK-GVDPATVLLSWHISRGSS 257
A.niger|NRRL3|10050_LarA        -------GVPLLVTRPEVKEVAESASKRLGT-TVTPAQVILAWSQVGGHS 266
P.rubens_Pc20g15580             -------GIPLLITRPEVKEVAESVAKRTGQ-EVSPAHVILAWSQVGGHS 266
A.nidulans_AN7193               -------NIPLLVAHPEVKAVAEEASKRLGK-TVTPAQVILNWSQEGGHS 266
A.oryzae_AO090023000264         -------GEPLLITRPEIKAVAEEAGKRLGK-EVSPAQVILAWSQVGGHS 265
N.haematococca|Necha2|36240     -------NIPLLFSHPDIKSLAERLSRERGQ-EVTPTQVLLSWAQSGGHS 270
P.nodorum_SNOG_02188.3          -------GLPLLVQHDTVKEVAK------AN-SATPAQVILAWAQVGGHS 260
N.haematococca|Necha2|100823    -------GLPLLVNVPEVKAIAERLSAAQGK-TVTPAQVVLAWSTLDGHL 263
T.reesei|Trire_Chr|110647       -------GLPLLVSSDEVKAVADNLSKKQGK-TVTPAQVILAWSQIGGHT 262
A.niger|NRRL3|7282              -------NIPRAVDDPLVIETAK------KL-NKTPAQVLISWAVQRGTV 252
S.cerevisiae|Sacce1|3067        --LQLAKTTPTLFENDVIKKVSQ------NHPGSTTSQVLLRWATQRGIA 272
C.boidini|Canbo1|6002           --HPKVKDCKTLFTHETITAIAS------AH-DVAPPKVLLRWATQRGLA 268
S.stipitis|Picst3|89614         --QGRALNTSPLFENETIKAIAA------KH-GKSPAQVLLRWSSQRGIA 266
```

```
N.haematococca|Necha2|38020      IFSKRAHGAEPLLEAELIKGFAD------KY-SKTPAQILLRWATQRGIA 271
V.alfalfa|Veral1|9347            AFNKRARGAQGLFEVESIKSLAG------KY-GVTPAQVLLRWATQRGVA 272
P.oryzae_MGG01404                --HPRVSKVEPLFTHPTIKAIAD------KH-GRTGAQVLLRWATQRDIV 275
V.alfalfa_|Veral1|801            --NPRAAAVPPLMEVPLVKAIAA------RH-GRTPGQVLLRWCTQRGIV 275
P.nodorum_SNOG_12824.3           --MKIAADTPLLFDHPVIKKIAE------KH-SKTPAQILLRWSTQRGLS 326
P.oryzae_MGG03648                --MEHAEALTPLLEEPTIVKLAE------KY-GKDPGQVLLRWATQRGLA 269
N.crassa_NCU08384                --MEHAQKLQPLLEDPTIKAIGD------KY-NKDPAQVLLRWATQRGLA 267
N.haematococca|Necha2|102983     --MDRAKDAVPLMQHEVFTTLAE------KY-GKTPAQVLLRWSTQRGIS 270
T.reesei|Trire_Chr|111218        --MPRAKSVAPLMDSPVIKALAD------KH-RRTPAQVLLRWATQRGIA 269
B.cinerea_BC1T12462              --WDHAQNTPPLFEHPDILKIAEKT-------KKTPAQVLLRWATQRGLA 269
P.rubens_Pc16g09600              --LKHAKDTPLLFEHATITSIAE------KH-GRTPAQVLLRWSTQRNVA 267
A.nidulans_AN0423                --VKQAEGAPPLFEHPVIKDIAE------KH-GKTPAQVLLRWATQRGIA 267
A.niger|NRRL3|1952_XyrA          --VQNAVDSPPLFEHQLVKSIAE------KH-GRTPAQVLLRWATQRGIA 267
A.oryzae_AO090003000859          --VKNAVDTPPLFEHNTIKSLAE------KY-GKTPAQVLLRWATQRGIA 267
B.cierea_BC1T00765               -------GKT-VIQNSELISIAE------KK-GVSIGQILIAWGIKRGYV 264
B.cinerea_BC1T04788              -------GEK-VSTNKDLNAIAE------KN-GASLAQVLIAWGLKRGYA 268
A.niger|NRRL3|2193               -------GEK-VSENQTLNEIAK------KG-GYTLAQVLIAWGLRRGYV 264
A.oryzae_AO090003001045          -------GER-VSENKTLNEIAQ------KG-GNTLAQVLIAWGLRRGYV 264
A.nidulans_AN5563                -------GER-VSENKTLNEIAQ------KG-GNTLAQVLIAWGLRRGYV 264
P.rubens_Pc22g04850              -------GER-VSENKTLNDIAE------KG-GNTLAQVLIAWGLRRGYS 264
P.oryzae_MGG00097                -------GER-VRDDPGLNAVAN------RS-NMTLAQALLGWGVKRGYV 267
N.haematococca|Necha2|64813      -------GEK-VRTNKTLNEVAD------RS-GHTLAQVLLAWGLRRGYS 265
P.nodorum_SNOG08193.3            -------GEQ-VRTNPKLNEVAQ------RS-GHDLAQVLLAWGLQRGYV 267
N.crassa_NCU04923                -------GER-VRDDPTLKAVAE------RS-GHNLAQVLLAWGLRRGYV 269
T.reesei|Trire_Chr|116623        -------GER-VRDNPTLKAVAE------RS-GYSLAQILLAWGLKRGYV 263
Clustal Consensus                                 .   .                 : :    .

                                        410       420       430       440       450
                                 ....|....*....|....|....|....|....|....|....|....|
A.nidulans_AN1274                VIPKSVSKSRIDANF-EI-DGWSLTDEEINEL----DNLKDR-------F 279
A.oryzae_AO090038000442          VIPKSVSKERIDTNF-EL-DGWNLTDEEVNQL----DNLKDR-------R 280
A.niger|NRRL3|10868_XyrB         VIPKSVSKSRIDANF-EL-DGWELTAEEIEQL----DNLKDR-------F 280
P.rubens_Pc16g05650              VIPKSVSKSRIEGNF-DI-NGWDLTDDEVNQL----DNLKDR-------F 279
B.cinerea_BC1T03301              VIPKSVNKERVEKNY-EL-DGWELTSEEVEKL----DNLKDR-------F 280
V.alfalfa|Veral1|1447            VIPKSVSKERIAANY-EL-DGWELTDDEIKQL----SSIPDR-------F 280
P.nodorum_SNOG_14959.3           VIPKSVTASRIQGNF-QL-DGWELTAEEMKEI----DSLPER-------F 280
N.haematococca|Necha2|73244      LLPKSVTKERIEANF-DL-RGWQLTDREMASL----SGIPDR-------F 280
N.crassa_NCU04510                VLPKSVTEERIKANI-DL-EGWSLTDEEIAQI----DEVHK-ENS----F 306
N.haematococca|Necha2|69859      VVPKSVTPSRIESNL-DL-DGWELTDDEMDML----DGIETR-------F 276
N.haematococca|Necha2|79737      VIPKSVTSTRIASNF-DL-DGWALSEDEIAEL----TGIKTR-------S 287
S.stipitis|Picst3|63050          VLPKSVTNSRIISNL-KT---FTLPEEDFEAL----NKLSEKDGI----H 282
C.boidini|Canbo1|3509            VLPKSVSSARIESNL-VV---VKLDDEDGEQI----NSIHETEGV----Q 292
S.cerevisiae|Sacce1|5773_GCY1    VLPKSVNPDRIKTNR-KI---FTLSTEDFEAI----NNISKEKGE----K 298
S.cerevisiae|Sacce1|1403_YPR1    VLAKSVNPERIVSNF-KI---FTLPEDDFKTI----SNLSKVHGT----K 298
C.boidini|Canbo1|1386            PLPKSVTESRIISNI-KI---IDLDEEVGGKI----DEISKIRGE----K 295
S.stipitis|Picst3|88249          VLPKSVTESRVQSNF-EV---INLSDEDGQTI----DNIHKVKGV----H 291
S.stipitis|Picst3|31015          VLPKSVSDSRIESNF-NV---VDLSDEDGEEL----NNLHKVKGI----K 293
A.oryzae_AO090010000381          VLPKSVKESRISSNS-QV---IPLSQEDMNVL----NGLAAQGKA----K 286
P.rubens_Pc12g04240              VLPKSISENRIVSNR-QV---IPLSREDMDVL----NGLAAQGKA----K 286
P.oryzae_MGG_02921               VIPKSVNPDRIKANA-QL---KDLDAEDMKLLNDYSEQLAKDGKL----N 293
N.crassa_NCU01906                VLPKSTNPERIEANA-KL---IELDAEDQKLLNDYSEGLVKEGKV----Q 293
N.haematococca|Necha2|67734      VLAKSVTPERITANK-TI---VDLDADDLKLLNDYSDDLTKKGEL----K 289
T.reesei|Trire_Chr|113861        VLAKSVTPARIKANL-EI---VDLDDEDMKLLNDYSNDLASKGEL----K 292
V.alfalfa|Veral1|4674            VLPKSTSTDRIKANL-QT---VKLDDEDLKSLNDYSADLAAKNEF----K 295
B.cinerea_BC1T02386              VLAKSVTLERIKANM-NI---VKLDDADMKILNDYSDDLKKNGKL----M 292
P.nodorum_SNOG07475.3            VLAKSVTPSRIDENR-KL---IQLDGTDMEKL----ESIHKTKGI----T 313
A.nidulans_AN5986                VLAKSVTPSRIEENR-KL---VKLEPEDVELIGKYSAELAATNGF----Q 297
A.oryzae_AO090011000614          VLAKSVTPSRIEDNR-KL---VQLDESDMATIAKYTDDLAARKAF----Q 295
P.rubens_Pc22g20340              VLAKSVTPARIEANRADL---IHLDAEDLATLRKYSDDLQAEGKL----Q 296
A.niger|NRRL3|6930               VLAKSVNPSRIEGNR-NL---VALDDADMATIAKYTNDLASKNAF----Q 299
A.niger|NRRL3|10050_LarA         VIPKSVTPSRIQENF-KE---VELTPEEIAKV----SELGKDR------R 302
```

```
                                 *
P.rubens_Pc20g15580             VIPKSVTPSRIRDNF-KE---IELTPEEVEKV----SALGQNR------Q 302
A.nidulans_AN7193               VIPKSVTASRIAENF-QE---VELTKEELAKV----SKLGEER------R 302
A.oryzae_AO090023000264         VIPKSVTPARIAANF-QE---IELTPEEIAKV----TALGTE-------R 300
N.haematococca|Necha2|36240     VIPKSVTPARIAANF-EE---IDLSADDLRVV----EGVGKEQ------R 306
P.nodorum_SNOG_02188.3          VIPKSVTASRIQENF-KE---IELSKEDFEKI----EEIGKKEP-----R 297
N.haematococca|Necha2|100823    VIPKSVTASRIRENF-QE---VELDAEAIKEL----DKLGEKP------Q 299
T.reesei|Trire_Chr|110647       VIPKSVTKARIAENF-QE---VELDDEAIAAL----NKLGEKP------Q 298
A.niger|NRRL3|7282              VLPKSVTPERIESNF-QD---FVLPDDAFSTI----QSLERH-------Q 287
S.cerevisiae|Sacce1|3067        VIPKSSKKERLLGNL-EIEKKFTLTEQELKDI----SALNAN-------I 310
C.boidini|Canbo1|6002           IIPKSNKKERLLGNL-TV-NDFDLTEEELEKI----ESLDIG-------L 305
S.stipitis|Picst3|89614         IIPKSNTVPRLLENK-DV-NSFDLDEQDFADI----AKLDIN-------L 303
N.haematococca|Necha2|38020     VIPKSNNPNRLAQNL-DV-VAFDLTSDELESI----SALDRG-------L 308
V.alfalfa|Veral1|9347           VIPKSNSQDRLKQNL-EV-NGFDLTAEELESI----SDLDRG-------L 309
P.oryzae_MGG01404               VIPKSNNVDRLKQNL-DC-VSFDLSDDEVKQI----SDLDCG-------V 312
V.alfalfa_|Veral1|801           VIPKSMRAERVRENL-DC-CGFDLEAGELEAI----SGLDRG-------L 312
P.nodorum_SNOG_12824.3          VIPKSNSQNRLQQNL-DV-TGFDMSESEIAEI----SDLDKN-------L 363
P.oryzae_MGG03648               VIPKSSREKTMKSNF-EA-VGWDMEDSDIKTI----SALDKG-------I 306
N.crassa_NCU08384               IIPKSSREATMKSNL-NS-LDFDLSEEDIKTI----SGFDRG-------I 304
N.haematococca|Necha2|102983    VIPKSTRLDLMLENL-AS-VSFNIDEEDMARI----SKMDLH-------L 307
T.reesei|Trire_Chr|111218       VIPKTSRPEVMAQNL-DN-TSFDLDSEDLAKI----ADMDLN-------I 306
B.cinerea_BC1T12462             VIPKSNNQSRLEQNLHVT--DFDLEKEDLNAIT----KLDRNLRFN---- 309
P.rubens_Pc16g09600             VIPKSNNPTRLAQNL-TV-TDFDLEASELEAI----SALDKG-------L 304
A.nidulans_AN0423               VIPKSNNPARLLQNL-DV-VGFDLEDGELKAI----SDLDKG-------L 304
A.niger|NRRL3|1952_XyrA         VIPKSNNPQRLKQNL-DV-TGWNLEEEEIKAI----SGLDRG-------L 304
A.oryzae_AO090003000859         VIPKSNNPTRLSQNL-EV-TGWDLEKSELEAI----SSLDKG-------L 304
B.cierea_BC1T00765              VLPMSSNEGRIKTNG-TL---VDLTEEEFEKM----NKVSEGNE-----T 301
B.cinerea_BC1T04788             VLPKSSNPGRIAGNA-KL---ITLSDEDFAAV----NKVAEGRA-----T 305
A.niger|NRRL3|2193              VLPKSSNPARIESNF-KS---IQLTDEEYEAV----NKVAEGRH-----F 301
A.oryzae_AO090003001045         VLPKSSNPARIESNF-KS---IELTDEDFEAV----NKVAEGRH-----F 301
A.nidulans_AN5563               VLPKSSNPARIESNF-KT---IELSDEDYEAV----NAVAKGRH-----F 301
P.rubens_Pc22g04850             VLPKSSNPKRIESNF-KS---IELNDADFEAV----NEVAKTHH-----C 301
P.oryzae_MGG00097               VLPKSSTPSRIKSNI-EV---PDLSEADYQDL----WKVANGRKP----T 305
N.haematococca|Necha2|64813     VLPKSSTPSRIESNF-QV---PTLSDADFEAV----EGVAKGRH-----T 302
P.nodorum_SNOG08193.3           VLPKSSTPKRIESNW-LI---PELSKEDFEAV----NEVAKGRH-----T 304
N.crassa_NCU04923               VLPKSSTPSRIESNF-QI---PVLRDEDFKAI----QEVAKGRH-----C 306
T.reesei|Trire_Chr|116623       VLPKSSTPSRIESNF-NI---PELSDEDFEAI----QQVAKGRH-----T 300
Clustal Consensus                :. :     :  *         :       :      .

                                       460       470       480       490       500
                                ....|....|....|....|....|....|....|....|....|....|
A.nidulans_AN1274               KVC------GDD------WLPIKVFFGDDE-------------------- 297
A.oryzae_AO090038000442         KVC------GDS------WLPIKVFFGDDE-------------------- 298
A.niger|NRRL3|10868_XyrB        KVC------GDD------WLPVKVFFGDDE-------------------- 298
P.rubens_Pc16g05650             KVC------GDA------WLPIKVFFGDDE-------------------- 297
B.cinerea_BC1T03301             KVC------GDA------WLPIKVFFGDDE-------------------- 298
V.alfalfa|Veral1|1447           KVC------GDA------WLPIKVFFGDDE-------------------- 298
P.nodorum_SNOG_14959.3          KVC------GDS------WLPIKVFFGDDE-------------------- 298
N.haematococca|Necha2|73244     KVA------DDS------WLPVRVFFGDDEEIDATRPVSM---------- 308
N.crassa_NCU04510               KVC------GDD------WLPVKIFFGAGDS------------------- 325
N.haematococca|Necha2|69859     KVV------GDG------FLPKKVFFGDDE-------------------- 294
N.haematococca|Necha2|79737     KVV------GDS------WMPIRVFFGDDE-------------------- 305
S.stipitis|Picst3|63050         RWA------DPS------WNDFST-------------------------- 294
C.boidini|Canbo1|3509           RFV------SPK------WG-VPVFD------------------------ 305
S.cerevisiae|Sacce1|5773_GCY1   RVV------HPN------WSPFEVFK------------------------ 312
S.cerevisiae|Sacce1|1403_YPR1   RVV------DMK------WGSFPIFQ------------------------ 312
C.boidini|Canbo1|1386           RLF------NRN------WG-IPVFD------------------------ 308
S.stipitis|Picst3|88249         RFI------NPN------WDPVVVFDADSKL------------------- 310
S.stipitis|Picst3|31015         RFV------SPN------WDPIDVYGED---------------------- 309
A.oryzae_AO090010000381         RIN------TPL------FG-WDLGFDDWYSQ------------------ 305
P.rubens_Pc12g04240             RIN------TPL------FG-WDLGFDDWYKQ------------------ 305
P.oryzae_MGG_02921              RYV------FPP------FG-TDFGFPDKVGQ------------------ 312
```

```
N.crassa_NCU01906                RYV------YPP------FG-VDFGFPDKS-------------------- 310
N.haematococca|Necha2|67734      RYV------YPP------FG-VDFGFPDKS-------------------- 306
T.reesei|Trire_Chr|113861        RYV------YPP------FG-IDFGFPDKS-------------------- 309
V.alfalfa|Veral1|4674            RYV------YPP------FG-IDFGFPDKS-------------------- 312
B.cinerea_BC1T02386              RYV------FPA------FG-VDLGFPDRKP------------------- 310
P.nodorum_SNOG07475.3            RYV------YPP------FG-VNCGFADKPDGIDLS-------------- 336
A.nidulans_AN5986                RYV------YPP------FG-VDFGFPDKS-------------------- 314
A.oryzae_AO090011000614          RFV------YPP------FG-VDFGFPDKS-------------------- 312
P.rubens_Pc22g20340              RFV------YPP------FG-VNFGFPDKQ-------------------- 313
A.niger|NRRL3|6930               RFV------FPP------FK-LDFGFPDKIGRV----------------- 319
A.niger|NRRL3|10050_LarA         RYNTPYVANTPR------WD-IDIFGEEEEKPAGHKVIV----------- 334
P.rubens_Pc20g15580              RYNTPYTANKPR------WD-IDIFGEPEEKPAGHKVILSV--------- 336
A.nidulans_AN7193                RYNTPYVANKPR------WN-INIFGEEDEKPAKHKVIV----------- 334
A.oryzae_AO090023000264          RYNVPYVANKPR------WN-INVFGEPEEAPADHKVIL----------- 332
N.haematococca|Necha2|36240      RFNIPYIANKPR------WD-INLFGDESEAPATHKIII----------- 338
P.nodorum_SNOG_02188.3           RFNIPYVANKPR------WP-VNIFNEPEEKEAPHKVIV----------- 329
N.haematococca|Necha2|100823     RFNIPT-TYDPK------WD-IDVFGDEKEKDATFQVVLKV--------- 332
T.reesei|Trire_Chr|110647        RFNIPY-TYKPR------WN-INLFNTEEEKAAAHTAVIKL--------- 331
A.niger|NRRL3|7282               RMN------FPA-----RIG-VDIFSEVGEESVRKSALAWAEQQRVLKAK 325
S.cerevisiae|Sacce1|3067         RFN------DPWTW---LDGKFPTFA------------------------ 327
C.boidini|Canbo1|6002            RFN------DPWTW----GYNIPTFI------------------------ 321
S.stipitis|Picst3|89614          RFN------DPWD-----WDKIPIFV------------------------ 318
N.haematococca|Necha2|38020      RFN------DPGFYL--PNYPLRIFA------------------------ 326
V.alfalfa|Veral1|9347            RFN------DPGFYL--PDHPLRIFA------------------------ 327
P.oryzae_MGG01404                RFN------DPADL----SPPIYIFD------------------------ 328
V.alfalfa_|Veral1|801            RFN------DPGVT---HGESVRIFA------------------------ 329
P.nodorum_SNOG_12824.3           KFN------APTNY----GIPCYVFA------------------------ 379
P.oryzae_MGG03648                RFN------QPANY--FSTDKLWIFG------------------------ 324
N.crassa_NCU08384                RFN------QPTNY--FSAENLWIFG------------------------ 322
N.haematococca|Necha2|102983     KFN------QPTNY--FPTEKLWIFA------------------------ 325
T.reesei|Trire_Chr|111218        RFN------KPTNY--FSANKLYLFG------------------------ 324
B.cinerea_BC1T12462              ---------NPTDYL--GTLHIFA-------------------------- 322
P.rubens_Pc16g09600              RFN------DPIAV----SLVCVEY------------------------- 319
A.nidulans_AN0423                RFN------DPPNY----GLPITIF------------------------- 319
A.niger|NRRL3|1952_XyrA          RFN------DPLGY----GLYAPIF------------------------- 319
A.oryzae_AO090003000859          RFN------DPIGY----GMYVPIF------------------------- 319
B.cierea_BC1T00765               RFV------DLKNT----FG-WEVFGDE---------------------- 318
B.cinerea_BC1T04788              RFV------NMKDT----FG-YNVWPEEK--------------------- 323
A.niger|NRRL3|2193               RFV------NMKDT----FG-YDVWPEETAKNLSA--------------- 325
A.oryzae_AO090003001045          RFV------NMKDT----FG-YDVWPEETAKNLSA--------------- 325
A.nidulans_AN5563                RFV------NMKDT----FG-YDVWPEETAKNISA--------------- 325
P.rubens_Pc22g04850              RFV------NMRDT----FG-YDVWPEETASGLSI--------------- 325
P.oryzae_MGG00097                RFV------DMKDT----FG-YDLWKESQ--------------------- 323
N.haematococca|Necha2|64813      RFV------NMKDT----FG-YNVWPEESLENGTAAA------------- 328
P.nodorum_SNOG08193.3            RFV------NMKDT----FG-YDVWPEEANGELKA--------------- 328
N.crassa_NCU04923                RFV------NMKDT----FG-YDVWPEESDGQLKQE-------------- 331
T.reesei|Trire_Chr|116623        RFV------NMKDT----FG-YNVWPEEE--------------------- 318
Clustal Consensus


                                 ..
A.nidulans_AN1274                -- 297
A.oryzae_AO090038000442          -- 298
A.niger|NRRL3|10868_XyrB         -- 298
P.rubens_Pc16g05650              -- 297
B.cinerea_BC1T03301              -- 298
V.alfalfa|Veral1|1447            -- 298
P.nodorum_SNOG_14959.3           -- 298
N.haematococca|Necha2|73244      L- 309
N.crassa_NCU04510                D- 326
N.haematococca|Necha2|69859      -- 294
```

N.haematococca|Necha2|79737	--	305
S.stipitis|Picst3|63050	--	294
C.boidini|Canbo1|3509	--	305
S.cerevisiae|Sacce1|5773_GCY1	--	312
S.cerevisiae|Sacce1|1403_YPR1	--	312
C.boidini|Canbo1|1386	--	308
S.stipitis|Picst3|88249	--	310
S.stipitis|Picst3|31015	--	309
*A.oryzae*_AO090010000381	--	305
*P.rubens*_Pc12g04240	--	305
*P.oryzae*_MGG_02921	--	312
*N.crassa*_NCU01906	--	310
N.haematococca|Necha2|67734	--	306
T.reesei|Trire_Chr|113861	--	309
V.alfalfa|Veral1|4674	--	312
*B.cinerea*_BC1T02386	--	310
*P.nodorum*_SNOG07475.3	G-	337
*A.nidulans*_AN5986	--	314
*A.oryzae*_AO090011000614	--	312
*P.rubens*_Pc22g20340	--	313
A.niger|NRRL3|6930	--	319
A.niger|NRRL3|10050_LarA	--	334
*P.rubens*_Pc20g15580	--	336
*A.nidulans*_AN7193	--	334
*A.oryzae*_AO090023000264	--	332
N.haematococca|Necha2|36240	--	338
*P.nodorum*_SNOG_02188.3	--	329
N.haematococca|Necha2|100823	--	332
T.reesei|Trire_Chr|110647	--	331
A.niger|NRRL3|7282	A-	326
S.cerevisiae|Sacce1|3067	--	327
C.boidini|Canbo1|6002	--	321
S.stipitis|Picst3|89614	--	318
N.haematococca|Necha2|38020	--	326
V.alfalfa|Veral1|9347	--	327
*P.oryzae*_MGG01404	--	328
*V.alfalfa*_|Veral1|801	--	329
*P.nodorum*_SNOG_12824.3	--	379
*P.oryzae*_MGG03648	--	324
*N.crassa*_NCU08384	--	322
N.haematococca|Necha2|102983	--	325
T.reesei|Trire_Chr|111218	--	324
*B.cinerea*_BC1T12462	--	322
*P.rubens*_Pc16g09600	--	319
*A.nidulans*_AN0423	--	319
A.niger|NRRL3|1952_XyrA	--	319
*A.oryzae*_AO090003000859	--	319
*B.cierea*_BC1T00765	--	318
*B.cinerea*_BC1T04788	--	323
A.niger|NRRL3|2193	--	325
*A.oryzae*_AO090003001045	--	325
*A.nidulans*_AN5563	--	325
*P.rubens*_Pc22g04850	--	325
*P.oryzae*_MGG00097	--	323
N.haematococca|Necha2|64813	--	328
*P.nodorum*_SNOG08193.3	--	328
*N.crassa*_NCU04923	--	331
T.reesei|Trire_Chr|116623	--	318
Clustal Consensus		

Multiple alignment of pentose reductases and related proteins. Conserved motifs for AKRs are indicated as: green boxes = active region motifs, red box = coenzyme binding motif, red asterisks = catalytic tetrad, blue asterisks = conserved Arg residue
