# Supplementary material for: Characterization of d-xylose reductase, XyrB, from Aspergillus niger
Source: Biotechnol Rep (Amst). 2021 Mar 15;30:e00610. doi: 10.1016/j.btre.2021.e00610 (PMC8020424; doi:10.1016/j.btre.2021.e00610)
Supplement: Supplementary file 2 [file mmc2.pdf]

Supplemental File S2. Purification parameters and results of XyrB.

Table S2-1 Protein purification parameters

Parameter	Value
Column Volume (CV)	0.962 mL
High Pressure Value	0.3 MPa
Flow Rate	1.0 mL/min
Elution Isocratic Volume	10 CV
Protein Detection	Absorbance at 280 nm

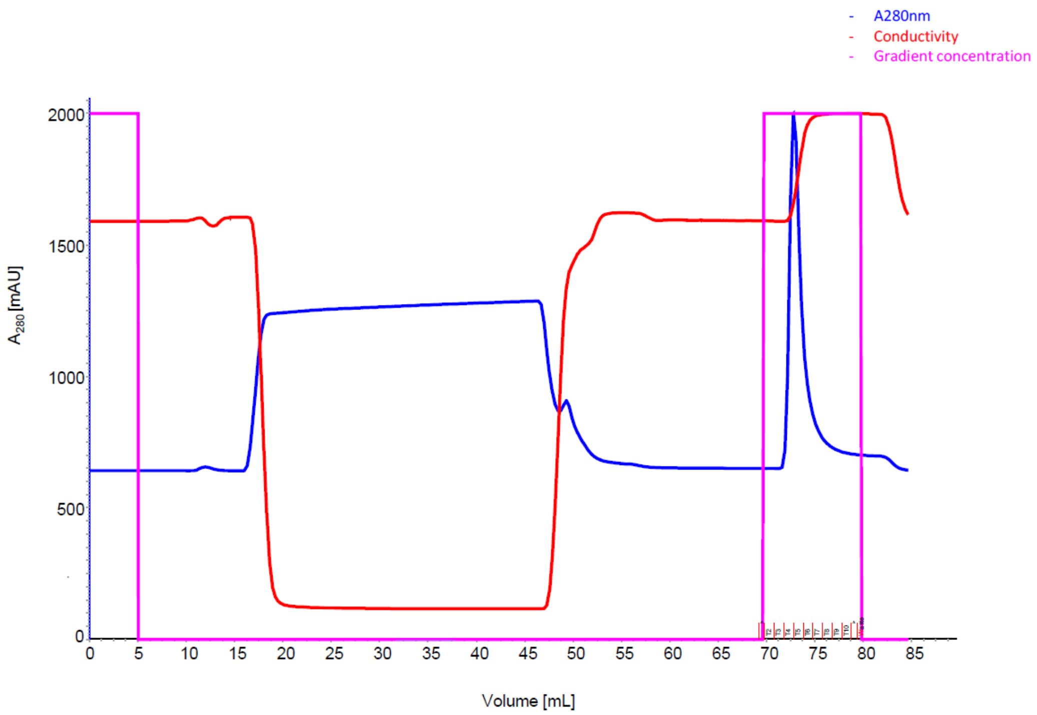

Figure S2-1. FPLC chromatogram showing the elution profile of XyrB purification.

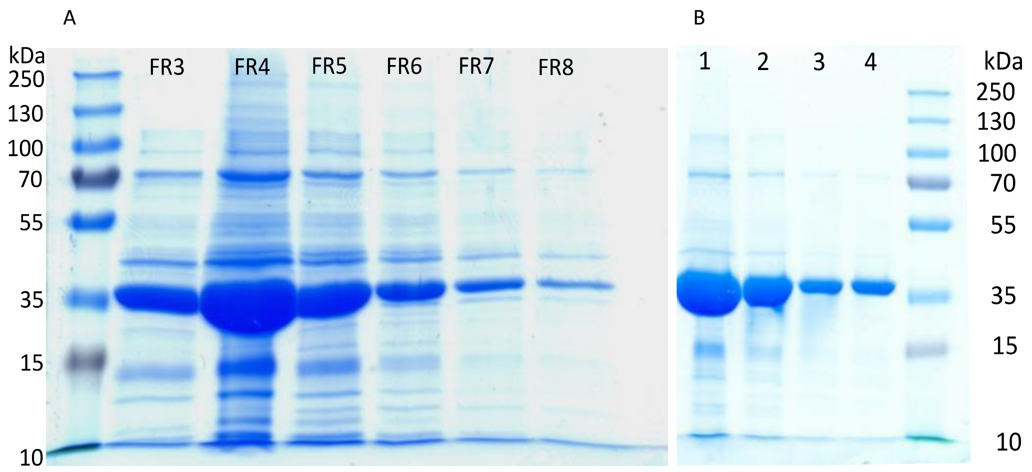

Figure S2-2. A- Collected fractions (FR 3-FR 8) corresponded to 280 nm absorbance peak containing XyrB; B- desalted XyrB dilutions: 1- 10 fold dilution; 2- 20 fold dilution, 3- 50 fold dilution, 4- 100 fold dilution.
